# Simulating impaired left ventricular–arterial coupling in aging and disease: a systematic review

**DOI:** 10.1186/s12938-024-01206-2

**Published:** 2024-02-22

**Authors:** Corina Cheng Ai Ding, Socrates Dokos, Azam Ahmad Bakir, Nurul Jannah Zamberi, Yih Miin Liew, Bee Ting Chan, Nor Ashikin Md Sari, Alberto Avolio, Einly Lim

**Affiliations:** 1 Department of Biomedical Engineering, Faculty of Engineering, University of Malaya, 50603 Kuala Lumpur, Malaysia; 2 Graduate School of Biomedical Engineering, Faculty of Engineering, University of New South Wales, Sydney, NSW 2052 Australia; 3 University of Southampton Malaysia Campus, 79200 Iskandar Puteri, Johor Malaysia; 4 Department of Mechanical, Materials and Manufacturing Engineering, Faculty of Science and Engineering, University of Nottingham Malaysia, 43500 Selangor, Malaysia; 5 Department of Medicine, Faculty of Medicine, University of Malaya, 50603 Kuala Lumpur, Malaysia; 6 Macquarie Medical School, Faculty of Medicine, Health and Human Sciences, Macquarie University, Sydney, NSW 2109 Australia

**Keywords:** Ventricular−arterial coupling, Left ventricle, Heart valve, Blood circulation, Computational modeling

## Abstract

Aortic stenosis, hypertension, and left ventricular hypertrophy often coexist in the elderly, causing a detrimental mismatch in coupling between the heart and vasculature known as ventricular−vascular (VA) coupling. Impaired left VA coupling, a critical aspect of cardiovascular dysfunction in aging and disease, poses significant challenges for optimal cardiovascular performance. This systematic review aims to assess the impact of simulating and studying this coupling through computational models. By conducting a comprehensive analysis of 34 relevant articles obtained from esteemed databases such as Web of Science, Scopus, and PubMed until July 14, 2022, we explore various modeling techniques and simulation approaches employed to unravel the complex mechanisms underlying this impairment. Our review highlights the essential role of computational models in providing detailed insights beyond clinical observations, enabling a deeper understanding of the cardiovascular system. By elucidating the existing models of the heart (3D, 2D, and 0D), cardiac valves, and blood vessels (3D, 1D, and 0D), as well as discussing mechanical boundary conditions, model parameterization and validation, coupling approaches, computer resources and diverse applications, we establish a comprehensive overview of the field. The descriptions as well as the pros and cons on the choices of different dimensionality in heart, valve, and circulation are provided. Crucially, we emphasize the significance of evaluating heart−vessel interaction in pathological conditions and propose future research directions, such as the development of fully coupled personalized multidimensional models, integration of deep learning techniques, and comprehensive assessment of confounding effects on biomarkers.

**Supplementary Information:**

The online version contains supplementary material available at 10.1186/s12938-024-01206-2.

## Introduction

Age is well-documented as an independent and nonmodifiable risk factor for the progression of hypertension (HTN) and cardiovascular diseases [1]. The prevalence of HTN has increased significantly with population aging [2]. With advancing age, the vascular system undergoes structural, mechanical, and functional modifications characterized by endothelial dysfunction, vascular remodeling (i.e. aortic dilatation, elongation, tortuosity, and wall thickening) and fibrosis, and increased arterial stiffness, eventually resulting in blood pressure (BP) elevation [3, 4]. The most common form of age-related HTN is isolated systolic HTN described by increasing pulse pressure (PP) due to a significant rise in systolic BP with no change or uniform decline in diastolic BP [5]. Under chronically elevated pulsatile loading caused by arterial stiffening, the left ventricle (LV) undergoes progressive changes in the structure and function (termed myocardial remodeling) at the expense of increased oxygen demand and reduced cardiac reserve, eventually giving rise to heart failure (HF) [6]. Pressure overload in hypertensive patients often leads to an increase in cardiac chamber stiffness, LV wall thickening (termed LV hypertrophy (LVH)), myocardial fibrosis, and impairment in cardiac diastolic function (termed diastolic dysfunction), as manifested by abnormal filling pattern and elevated filling pressure. Diastolic HF, also known as HF with preserved ejection fraction (EF), is the most prominent hemodynamic dysfunction in aging [7]. Moreover, aortic and mitral valve pathologies with aortic valve stenosis (AS), as one of the most common and serious valve diseases, is often associated with aging and HTN [8]. The pathogenesis of AS incorporates cumulative calcification and fibrosis together with gradual reductions in valve area [9]. The underlying mechanisms will ultimately contribute to structural alterations and functional deterioration of the cardiovascular system which causes a mismatch in the coupling between the heart and vasculature. Thus, understanding their inter-relationship can offer critical mechanistic insights into how the complex cardiovascular system adapts with aging in association with or without other pathological conditions.

The interaction among LV function and systemic arterial (SA) properties, termed ventricular−arterial (VA) coupling, is well established as a major determining factor of global cardiovascular performance and efficiency [10]. This notion arises due to the heart and arterial tree being interconnected structures in terms of anatomy and physiology, and should be evaluated as a whole system [11]. VA coupling is usually estimated as the ratio of effective arterial elastance (Ea; arterial load index) to ventricular end-systolic (ES) elastance (Ees; LV contractility index) via echocardiography [12]. Age-related structural and functional changes in arterial properties will lead to a gradual rise in Ea while there is a compensatory surge in Ees due to LV remodeling (LVR), marking the progression toward a less effective system with preserved coupling (i.e. limited exercise capacity) [13, 14]. If there is a decline in Ees due to decreased in pump performance (e.g. LV systolic dysfunction), an increase in Ea will drastically cause VA decoupling. However, Ea does not provide a complete representation of pulsatile arterial load, which is a key determinant of cardiovascular function, that extensive analyses of pressure flow relations are required for substitution [15]. Besides, this interaction can also be characterized and quantified based on the novel measurement of arterial and myocardial function markers [16]. Cardiac structure and function, global and regional strain, LV mass index, relative wall thickness, systolic and diastolic function, and presence of myocardial fibrosis can all be assessed using echocardiography and cardiovascular magnetic resonance to determine cardiac remodeling and HF. In terms of vascular function, pulse wave velocity (PWV) can be measured using arterial tonometry, Doppler or flow magnetic resonance imaging (MRI) to indicate arterial stiffness. Biomarkers such as augmentation index (AIx), central aortic BP, systematic arterial compliance, aortic distensibility, and valvulo-arterial impedance (Zva) can also be employed to assess arterial function. For instance, the ratio of carotid−femoral PWV (as a gold standard for quantifying aortic stiffness) to global longitudinal strain (as a gold standard for evaluating LV performance) has been proposed to describe VA interaction in HTN [17]. Another novel method of assessing VA coupling is myocardial work index which can derived from the LV pressure—strain loop using speckle tracking echocardiography [18].

Despite the potential use of the proposed indicators as independent prognostic biomarkers in arterial HTN, they have not found wide acceptance in clinical use due to the difficulty in defining normal ranges for different patient cohorts and contradictory outcomes in the presence of uncontrolled confounding variables [19, 20]. These markers, measured at specific locations, are affected by other factors as a result of hemodynamic coupling [21] which impede their actual translation into clinical practice. For example, PWV, which is the current gold standard method for assessing arterial stiffness, is affected by LV ejection time [22], heart rate (HR) [23–25] and peripheral resistance when arterial stiffness remains unchanged [26]. LV ejection time denotes the duration from aortic valve (AV) opening to closure and represents the systolic phase during which the LV expels blood into the aorta. The inherent properties of the arterial tree are difficult to characterize in the presence of AS due to complications in the VA uncoupling relationship [8]. In order to utilize the proposed prognostic indicators in routine clinical practice, there is a need for in-depth understanding of how these are affected by intrinsic properties of the various cardiovascular components.

Computational models, established on the basis of sound physical and mathematical principles, are widely applied to study the ventricular, valvular, and vascular system due to the fact that simulations can be conducted in a more reproducible manner by tuning required parameters as compared to clinical settings. The development of computational models allows for a greater understanding of cardiac function by reproducing cardiovascular features observed experimentally that can provide meaningful insights into the interactions between different components of the cardiovascular system [27]. The modeling approach of integrating multiple diagnostic data obtained from different clinical modalities (e.g. echocardiography, MRI, BP measurements) not only allows for better understanding of disease extent in individual patients, but also facilitates the computation of hemodynamic variables that are hard to measure experimentally, such as myocardial stroke work (SW). However, most models tend to emphasize either the vasculature [26, 28–31] or the heart [32] without taking into account the effect of VA interaction. Hence, the aim of this systematic review is to evaluate the current state-of-the-art of computational modeling and simulation of the cardiovascular system in investigating the effect of impaired VA coupling in aging and disease on cardiovascular structure and function parameters. This paper is organized as follows: (i) model structures, including heart chambers, valve models and circulatory models in different dimensionalities; (ii) boundary conditions (BCs) for mechanical problem; (iii) model parameterization and validation; (iv) model coupling approach and the use of computational resources; (v) model applications in investigating ventricular, valvular and arterial diseases and (vi) future perspectives.

## Model structures

**Fig. 1:**
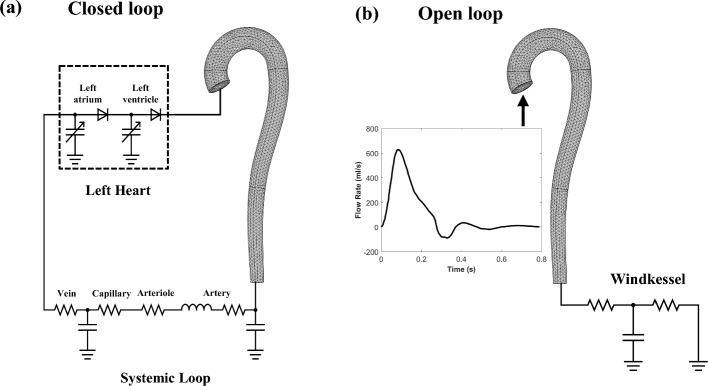
Comparison between **a** closed [70] and **b** open-loop multidimensional cardiovascular models comprised of a 3D idealized aortic geometry model coupled to 0D cardiovascular components

**Fig. 2:**
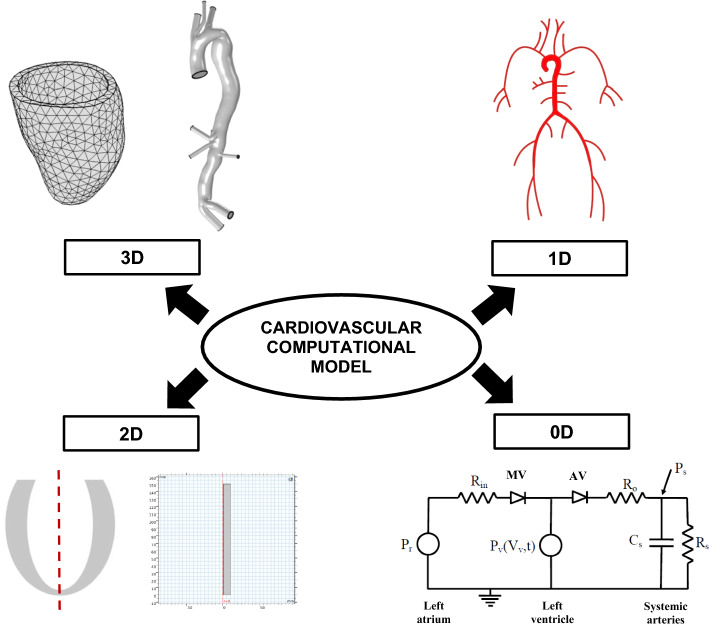
Different dimensions of cardiovascular modeling that include 3D LV and aorta adopted from [71], 2D axisymmetric LV and vessel, 1D SA, and 0D LV coupled to systemic circulation adapted from [72]

**Fig. 3:**
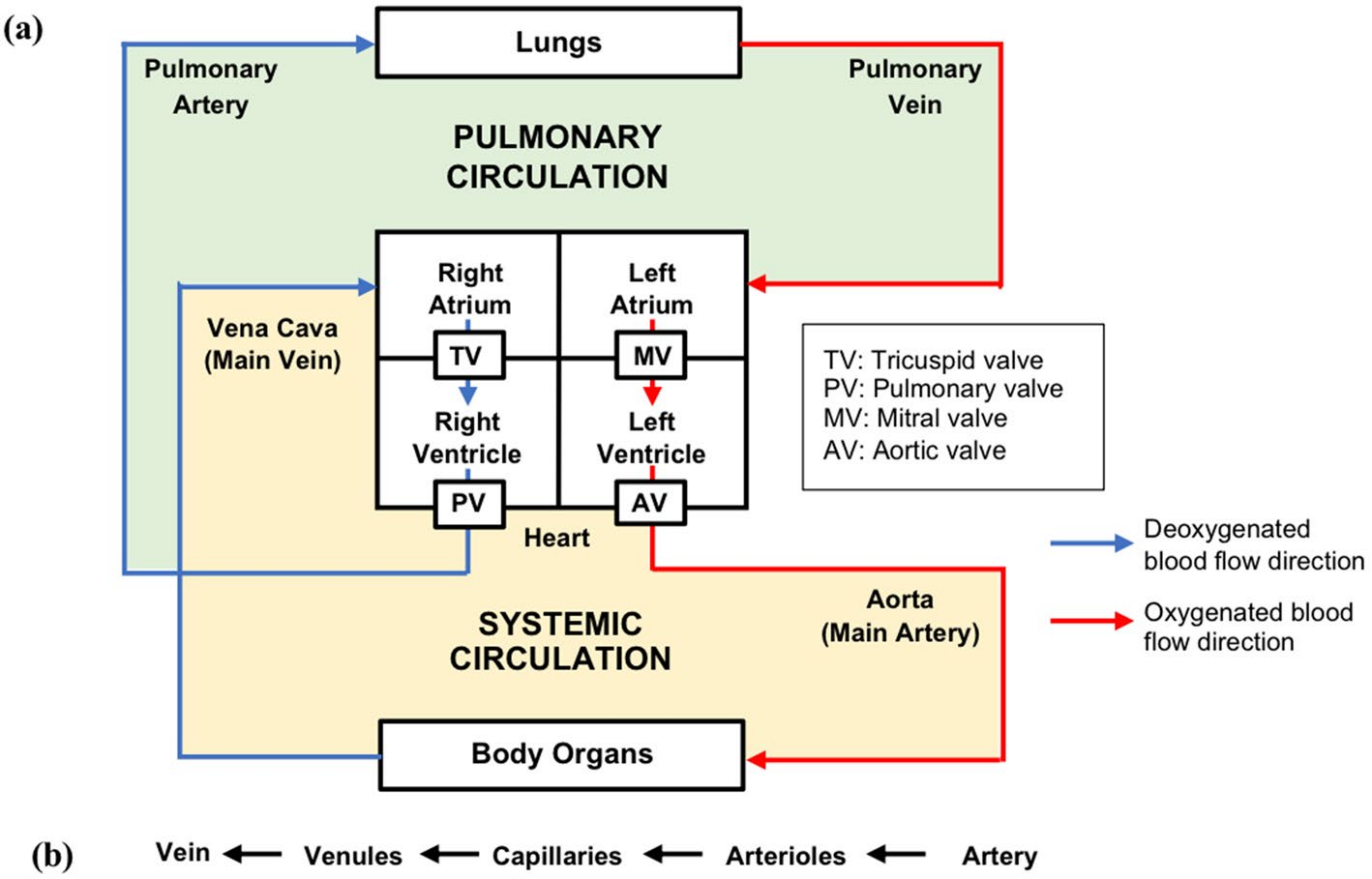
Block diagram of the cardiovascular system consisting of **a** heart, valves, pulmonary and systemic circulations and **b** blood flow path within blood vessels to and from the heart

**Fig. 4:**
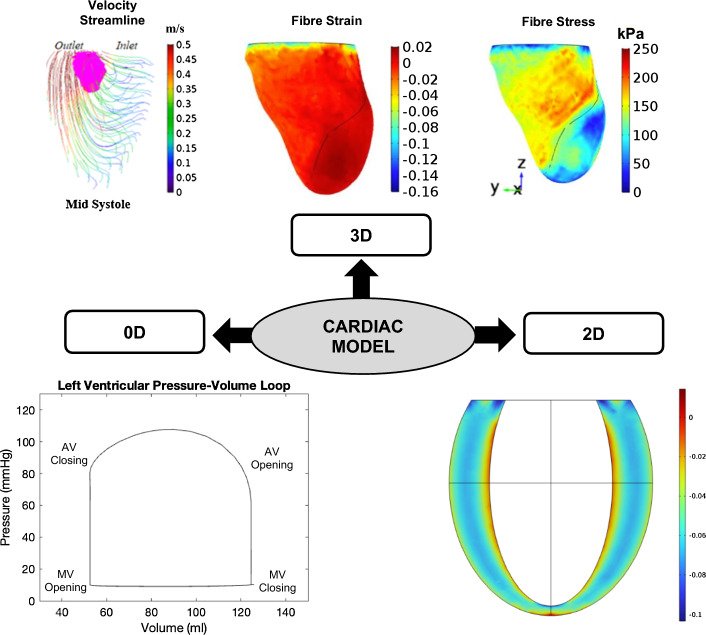
Simulation outcomes of cardiac models that can be visualized in different dimensions: (1) 3D FSI model shows vortex formation in LV outflow tract view with the 3D vortex is visualized by the magenta isosurface and the color map represents velocity magnitude of the velocity streamline (m/s) adopted from [71]; 3D electromechanical finite element model displays end-systolic fiber strain and stress distributions at the LV subendocardium adopted from [73]; (2) 2D axisymmetric LV model demonstrates strain distributions through the heart wall; (3) 0D time-varying LV elastance model illustrates a ventricular pressure–volume relationship [72]

**Fig. 5:**
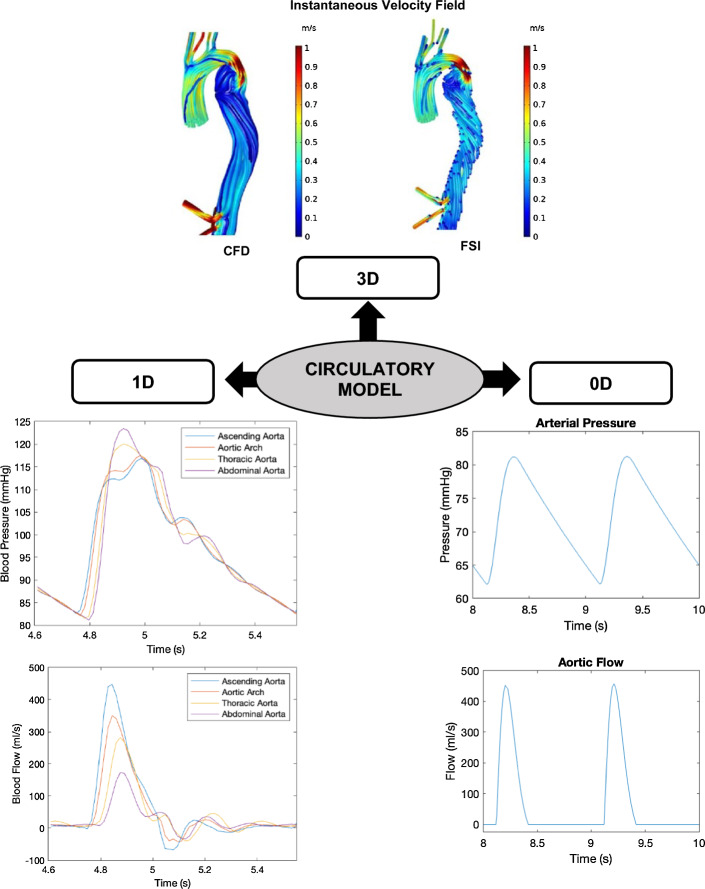
Simulation outcomes of circulatory models that can be visualized in different dimensions: (1) 3D CFD and FSI aortic model of a patient with type B aortic dissection shows the instantaneous blood velocity field at peak systole adopted from [74]; (2) 1D 55-segment SA model demonstrates blood pressure and flow waveforms at different locations from the ascending to the abdominal aorta produced using PWPSim [75]; (3) 0D lumped parameter model of the systemic circulation provides central aortic pressure and flow profiles produced based on [72]

To study VA coupling in aging and cardiovascular disease, numerous computational models have been developed, ranging from the simplest lumped parameter zero-dimensional (0D) models to more complex multidimensional models. Multidimensional modeling incorporates the benefits of different dimensional models, allowing both local and global hemodynamic information to be obtained with reasonable computational cost and higher accuracy. Open-loop (OL) models [33–46], which prescribe a constant filling pressure to the LV, as well as closed-loop (CL) models [47–66], which take into account the effect of venous return (filling pressure) on the heart chambers, have been proposed. Figure 1 illustrates the difference between OL and CL configurations of cardiovascular models. Meanwhile, the models can also be categorized into 0D, 1D, 2D or 3D (Fig. 2). 0D models are composed of a set of lumped parameter ordinary differential equations (ODE) used typically to represent hydraulic circuits [67]. 1D models incorporate a single spatial independent variable in addition to time, written as a set of partial differential equations (PDE) [45, 68], whilst 2D models incorporate 2 spatial variables plus time, and can be used to represent 3D axisymmetric geometries of the heart or blood vessels using cylindrical coordinates [65]. Finally, 3D models incorporate 3 spatial independent variables (in addition to time) and are usually employed to depict anatomically detailed features of the heart and blood vessels [64, 69]. Figure 3 illustrates the general layout of the cardiovascular system to be modeled. Regardless of their complexities, all VA coupling models comprise three components: the heart chambers (Fig. 4), the valves, and the circulatory system (Fig. 5). In the following section, a more detailed description of each of these components is provided.

### Heart chambers

#### 2D or 3D LV models

While LV mechanical [50, 51, 64–66] and electromechanical (EM) models [33, 48] have been commonly applied to assess myocardial wall stress and strain, computational fluid dynamics (CFD) [52] and fluid structure interaction (FSI) models [40] have been used to investigate fluid dynamics, such as vortices and energy losses. 3D LV models either use an idealized geometry (e.g. half prolate ellipsoid) [64] or patient-specific geometries reconstructed from medical images such as MRI [33, 40] or computed tomography (CT) [50]. Apart from the active LV region, several 3D geometries [33, 40, 50] also extend to the valvular and inflow/outflow tracts. To reduce computational cost associated with 3D simulation of the heart, Syomin et al. [65] modeled the LV as a 2D axisymmetric geometry.

##### Active and passive mechanical models

LV finite-element (FE) mechanical models [66] consist of equations describing active and passive mechanics whilst EM models have additional equations describing cardiac electrophysiology and action-contraction coupling [33, 48]. The most common method of modeling cardiac electrophysiology in humans utilizes the Ten−Tusscher−Panfilov ionic model [76, 77], which describes the dynamics of ionic fluxes across the cardiomyocyte membrane, coupled with a reaction−diffusion monodomain PDE to generate myocardial electrical propagation, which serves as the trigger for active stress generation [48, 78]. Active cardiac stress is normally modeled as a function of activation time, length (sarcomere)-dependent calcium sensitivity as well as maximal isometric tension, which depends on the intrinsic contractility [33, 79]. For simplicity, some models [50, 51, 64, 66] do not incorporate cardiac electrophysiology, but instead adopt a time course of contraction (or time-varying elastance (TVE)) uniformly across the entire ventricle and length-dependent calcium sensitivity to generate time-varying active stress profiles [80, 81]. While most active stress models incorporate the Frank−Starling mechanism through length-dependent force generation, the dependence on fiber velocity is neglected [33, 48]. Notably, a recently published paper [82] has highlighted the critical role of fiber-stretch-rate feedback in regulating blood flow ejected by the ventricles. In terms of passive cardiac mechanics, the most commonly-used constitutive model [33, 48, 83] is the Guccione-type models [84, 85]. Several models [50, 51] have also used the Holzapfel and Ogden anisotropic hyperelastic model [86] while Shavik et al. [64] used the Fung-type strain energy function [85] to model passive cardiac mechanics. As it is difficult to acquire patient-specific myofiber orientation due to long acquisition and reconstruction times, and motion artifacts in diffusion tensor MRI, the majority of 3D LV models simply adopt a rule-based approach for cardiac fiber orientation [87, 88], such that it varies linearly from around -60° at the epicardium to around +60° degrees at the endocardium.

##### CFD and FSI models

Due to the high computational cost, very few VA coupling models have adopted either CFD or FSI approaches. The CFD LV model developed by Zuo et al. [52] used a 3D velocity function (i.e. axial, radial and circumferential) to specify contraction, expansion, and twisting movements of the LV wall. Approximate solutions for the pressure and velocity fields were obtained by solving the fluid continuity equation coupled with the Navier−Stokes equations (NS) which are a set of PDEs that described the fluid substances motion. To more accurately model LV flow dynamics, the k−ω shear stress transport turbulence model has been proposed to replace the simpler laminar flow setting [52]. Although NS equations are more accurate in describing complex fluid flows, they are computationally expensive. Reynolds-averaged NS equations which are the simplified NS equations by time-averaging the flow variables provide a computationally efficient alternative for simulating turbulent flow. Another turbulence modeling approach utilizes the Large Eddy Simulation (LES) models within the NS equations [89], which offer improved accuracy in capturing unsteady flow compared to the Reynolds-averaged NS models at the expense of a higher computational demand. Only one selected study [40] adopted the FSI approach, which takes into account the interaction between the myocardium wall and the blood flow velocity. The solution for the model was obtained through a combined immersed boundary FE method.

#### 0D LV models

While 3D LV models provide detailed insights into cardiac dynamics, their complexity makes model personalization (calibration) difficult. Reduced-order models, made up of a set of lumped parameter ODEs, have therefore been developed to represent the LV cavity, especially when global hemodynamics such as pressure and volume are of interest. Several studies [37, 53, 60] modeled cardiac contraction based on a modified Hill model [90], which describes sarcomere mechanics using a contractile element arranged in series with an elastic element, which in turn is connected in parallel to a passive elastic element. The contractile element describes stress generation due to muscle activation, with its magnitude dependent on its velocity, length and activation function, while the passive elastic element describes passive stress due to muscle length change. Another study [34] adopted a microscopic Huxley-like model of actin−myosin binding [91] to generate myocardium active stress with a macroscopic LV cavity (i.e. chamber) deformation formulation. Unlike Hill’s model, the Huxley model considers dynamics of the filaments within muscle along with the probability of establishing cross-bridges between myosin heads and actin filaments. The resultant LV cavity volume is derived from myofiber stretch, while the LV cavity pressure is derived from myofiber stress and stretch, with an assumption that the myofiber stress is homogeneously distributed within the myocardial wall. On the other hand, the majority of 0D LV models which formulate VA coupling [35, 36, 38, 39, 41–47, 49, 54–59, 61–63] have adopted the simplest TVE model originally proposed by Suga et al. [92], which relates pressure and volume of the ventricle using a time activation profile. The pressure–volume (PV) relationship of the heart changes throughout a cardiac cycle following a TVE curve. The modified Hill's model allows for accurate prediction of force–velocity relationships in the LV, the Huxley-like model provides insights into the actin−myosin binding kinetics, and the TVE model accounts for changes in ventricular contractility over time.

### Valve models

The majority of VA coupling models [38, 46, 48, 54, 63] use a simple diode-like formulation to model cardiac valve dynamics, with instantaneous opening and closing determined by the pressure gradient across the valve. Several studies [50, 51, 61, 62] modeled the valves as a simple resistance element in the fully open or fully closed state neglecting opening and closing processes, while another study [34, 52, 64, 66] combined a diode with a linear or nonlinear resistance to capture the ideal characteristic of unidirectional flow in the heart valve, with Syomin et al. [65] including additional inductances and capacitors. Maksuti et al. [59] simulated the valves as small resistance and inertance. However, under normal or pathological conditions, valves can exhibit complex leaflet motions and flow dynamics that cannot be captured by such a simplified valve model. Most studies [33, 36, 37, 39, 41–44, 49, 53, 55, 58, 60] have thus modeled the AV based on the Bernoulli equation, where the instantaneous net pressure across the AV is expressed as a function of instantaneous flow rate, fluid inertance and the energy loss coefficient, which in turn depends on the effective orifice area (EOA) and aortic cross-sectional area at the sinotubular junction [93]. Neglecting inertial and turbulence losses at the AV, a key component of VA coupling, would compromise the precision of aortic pressure and flow wave shapes in both physiological and pathological conditions. In order to model the smooth opening and closing dynamics of the valves, Caforio et al. [33] considered that the effective aortic cross-sectional area changes with time and is dependent on the rate of valve opening and closure. Although valve motion is known to be influenced by various factors, the opening and closure rate is assumed to be determined by only two aspects: (i) the immediate pressure disparity across the valve and (ii) the present condition of the valve. A more advanced valve model was adopted by Laubscher et al. [47], which took into account valve cusp thickness, cusp heights, valvular opening angle, and the instantaneous valve flow rate to represent their valve pressure loss and motion models in determining the time-dependent flow coefficients of the valve.

### Circulatory models

#### 3D models

3D models of the arteries are able to provide detailed descriptions of local blood flow field or stress distribution in the wall. However, since they require complex anatomical and mechanical information and are computationally expensive, such models are normally used to simulate local hemodynamics of specific arterial sites of interest instead of the whole arterial tree.

##### Finite element models

As pathological remodeling and aging are commonly associated with changes in aortic microstructure, Shavik et al. [64] developed a FE model of the thoracic aorta to investigate the separate contributions of key load bearing constituents, i.e. elastin, collagen fibers and smooth muscle cells on its mechanical behavior (e.g. pressure−diameter relationship) and VA coupling. Stress in the aortic wall was derived by summing the strain energy functions related to the main tissue constituents, including the elastin-dominated matrix, collagen fiber families, and vascular smooth muscle cells, each characterized by different constitutive parameters and mass fractions.

##### CFD and FSI models

Two selected studies [37, 49] utilized CFD analysis to investigate detailed blood flow dynamics, such as wall shear stress (WSS), oscillatory shear index (OSI) and kinetic energy, in different disease scenarios (including ascending aorta thoracic aneurysm (ATAA), mitral valve (MV) disease and aortic coarctation (COA)). In both models, 3D patient-specific anatomies of the thoracic aorta were reconstructed based on computed tomography angiographs (CTA). While Cosentino et al. [37] assumed laminar blood flow, Sadeghi et al. [49] adopted a 3-D Lattice−Boltzmann CFD approach using LES to simulate blood flow through the aorta. LES is suitable for modeling turbulent vascular flows and physiological low-Reynolds transitional flow, which commonly occurs under pathophysiological conditions. To further explore the impact of pressure on the aortic wall stress, Cosentino et al. [37] adopted an one-way FSI approach, where pressure load forces at each node of the aorta wall were exported from the CFD results in FLUENT (ANSYS Inc, Canonsburg, USA) into a FE model developed in ABAQUS (SIMULIA Inc, Providence, USA). The mechanical behavior of the aortic wall was characterized using the anisotropic hyperelastic Holzapfel–Gasser–Ogden material model [94].

#### 1D models

In order to avoid computational load associated with the 3D models, 1D models of the large arteries are normally used when local vascular changes, such as tapering, branching or stenoses, are being investigated, or when the influence of physiological and disrupted wave transmission on the circulation is under study. 1D models of blood flow typically comprise the one-dimensional continuity and momentum equations based on the NS equations, coupled with a constitutive law of the arterial wall, which links the change in the pressure to the wall deformation and/or deformation rate. While several studies [40, 45, 57, 58] have modeled the arterial wall as linear elasticity, majority of the VA coupling models [33–36, 39, 41, 54, 56] have adopted nonlinear viscoelastic constitutive law for the arterial wall, which inhibits nonphysiological high frequency oscillations in the simulated aortic pressure waves. Different numbers of arterial segments have been used in the 1D models, including 1 (aorta) [34], 24 [40], 55 (main SA) [45, 56–58] referring to Wang and Parker et al. [95], 103 (including 55 large arteries, coronary circulation and the circle of Willis) [35, 36, 39, 41] according to Reymond et al. [96], 116 [33] or 128 arterial segments (involving central vessels and major peripheral arteries) [54] based on Avolio et al. [97].

#### 0D models

Reduced-order models, such as the 0D lumped parameter models of the circulation, are frequently employed when global hemodynamic parameters such as flow and pressure are of interest. The majority of VA coupling models [38, 42–44, 46–52, 54–66] have modeled the systemic and/or pulmonary circulations based on Windkessel (WK) models in a single- or multicompartment configuration. For single-compartment models, Garcia et al. [44] used a 3-element WK model consisting of a capacitance (i.e. compliance), characteristic impedance, and peripheral resistance, whilst other studies [59, 61–63] also added an inductance to represent fluid inertance in the large arteries to form a 4-element WK model [98]. In multicompartment models, a series of single-compartments with a combination of either a resistance, inertance and/or compliance are implemented to represent different elements of the circulation (i.e. artery, arteriole, capillary, venule, and vein). In addition to representing the entire circulation, 3-element WK models have also been employed to represent the portion of the circulation downstream of a higher-order model and to identify the connection between pressure and flow at its boundaries [33–37, 39, 41, 45]. In order to allow an explicit parameterization of the geometrical and mechanical properties of the distal arteries/arterioles, as well as microvascular effects, two studies [40, 56] coupled structured-tree (ST) models proposed by Olufsen et al. [99, 100] to each terminal vessel in a 1D network of large arteries as outlet BCs. In such ST models, each parent artery in the vascular bed bifurcates into daughter arteries with smaller radii, and the bifurcation process persists until the daughter vessel radius reaches a minimum threshold. Despite the advantages of the ST model that can provide more accurate flow and pressure predictions, it is computationally more complex compared to the WK model [101].

## Boundary conditions

Prescribing appropriate BCs is a critical aspect in modeling complex mechanical and hemodynamic behaviors, such as cardiac motion, accurately. In 2D/3D LV models, the standard configuration for the structural domain includes three boundaries: the endocardium, the epicardium, and the ventricular base, which delineates the artificial boundary where the LV geometry is intersected. At the endocardial surface, the most commonly applied BC is the normal stress, which accounts for the pressure exerted by the blood within the cardiac cavity [33, 40, 48, 65]. For simplicity, the ventricular base is typically fully constrained from longitudinal movement (i.e. shortening or lengthening along the apex-base direction), thus allowing only in-plane motion, while leaving the remaining myocardial boundaries unrestricted [40, 64, 65]. In terms of in-plane motion, Chen et al. [40] and Syomin et al. [65] only allowed radial wall motion (i.e. contraction and expansion), while Shavik et al. [64] allowed additional circumferential displacement (i.e. twisting and untwisting) with the epicardial−basal edge fixed. Although segments of the inflow and outflow tracts were integrated into the LV geometric model, they only served as BCs with assumed rigidity, alongside the fixed inlet and outlet annuli [40, 50]. This setup may lead to an overestimation of apical motion as there is a lack of constraint in this region [40].

On the other hand, several published cardiac models have taken into account the influence of the pericardium in constraining and guiding heart dynamics. Veress et al. [66] introduced a soft tether mesh around the LV model to represent tissues surrounding the myocardium, with its edges completely constrained to eliminate rigid body motion. Caforio et al. [33] utilized spatially varying, normal, spring-type BCs at the epicardial wall using a Robin (i.e. mixed-type) BC to mimic the effect of the pericardial constraint on the LV wall. The spring stiffness was gradually scaled, with a maximum stiffness value, transitioning from zero at the base to one at the apex. Meanwhile, Regazzoni et al. [48] introduced generalized spring-damper element in both normal and tangential directions at the epicardial wall to constrain rigid ventricle rotation around the apico-basal axis while preserving torsion. With regards to the ventricular base, Caforio et al. [33] incorporated a portion of the aorta and employed omni-directional springs at the clipped aortic rim to address basal movement. On the other hand, Regazzoni et al. [48] imposed energy-consistent BCs at the ventricular base to account for the effect of the neglected part over the basal plane [102, 103]. This formulation is crucial when the 3D mechanical heart model is coupled with the lumped parameter circulation models as it enables accurate replication of the downward movement of the atrioventricular plane during the ejection phase [104, 105]. Furthermore, Caforio et al. [33] included additional springs on the septum in the LV model to prevent nonphysiological rotation. In a healthy individual, the LV generally undergoes a twisting motion between apical and basal regions of around 15 degrees, shortens from the base towards the apex by approximately -15% to -20%, and experiences wall thickening of about 30–40% during systole [106].

In terms of the 3D aortic model, the geometry typically encompasses the aortic root or ascending aorta and extends down to the descending aorta. Most studies have also included the supra-aortic branching vessels (i.e. brachiocephalic trunk, left common carotid artery, and left subclavian artery). The simplest method of constraining the aorta geometry involves immobilizing the distal ends of the supra-aortic vessels, the AV, and the descending aorta, in all directions [37]. To address constraints from the surrounding tissues and organs on the aorta, additional BCs were implemented on the outer arterial wall [107]. For example, mechanical tethering of the aorta to the spine was simulated by modeling the intercostal arteries as vessel stumps with structural Dirichlet conditions along the aorta [107]. In another study, a viscoelastic material representing external tissue surrounding the aorta was applied on the aortic wall using a generalized Robin BC [108]. Aortic root motion was accommodated using stiff springs at the proximal end, while the movement of the distal ends of the branching vessels and the descending aorta were constrained using spring-damper mechanisms [108, 109].

## Model parameterization and validation

Most model parameters in the selected studies were either obtained from the published literature [34–36, 38, 41, 43–45, 48, 51–55, 57–60, 64–66] or derived based on population-averaged hemodynamic data in a healthy or diseased cohort [39, 42, 46, 47, 56, 61–63]. Only a few VA models [33, 37, 40, 49, 50] parameterized their models based on subject-specific data, in which a subset of the model parameters was fitted to subject-specific measurements, by applying various parameter optimization methods and robust inverse problem strategies. Common optimization techniques include nonlinear least-squares algorithms such as Levenberg–Marquardt [33, 66, 110, 111] and trust-region-reflective approaches [40, 49, 112] to iteratively adjust parameters by minimizing the least-squares differences between the observed data and the model output. Prior to this, it is necessary to establish initial parameter values, often sourced from published literature, to initiate forward simulations to reach steady state, while the final parameter values are obtained through successive approximations and comparisons. Arguably, a properly personalized model could more accurately predict physiological or pathological status [113]. To validate the model, comparison with single- or multimodality measurements were performed [37].

Due to the limited availability of clinical and experimental measurements, a local sensitivity analysis is required to determine the subset of model parameters to be optimized for subject-specific simulations. Such a sensitivity analysis assesses the impact of individual model parameters on the output hemodynamics quantities of interest, one at a time (keeping all other model parameters constant). Gul et al. [38] has further proposed Sobol’s method, a variance−decomposition method used for global sensitivity analysis, to quantify the impact of model parameters and their interactions on the output quantities of interest. The analysis was performed over the entire feasible region of model parameters, with parameter distributions estimated using published medical data and expert opinions.

In terms of the LV model, the main model parameters are those relating to active contractility, passive stiffness and TVE profile. These parameters are optimized by minimizing the differences between computed and measured LV pressure and volume changes during the systolic and diastolic phases, respectively. As the gold standard measurement of LV pressure involves invasive catheterization, it is not commonly performed [50] and the data is usually taken from previous studies [46, 61]. Instead, systolic LV pressure is estimated from the summation of brachial cuff pressure and transvalvular pressure gradient, which is in turn estimated from Doppler-ultrasound flow measurements based on Bernoulli’s principle [49]. On the other hand, changes in LV volume over a cardiac cycle, intracardiac chamber flow, blood flow velocity at the valves as well as myocardial velocity, are all obtained using Doppler echocardiography or MRI techniques (cine MRI and 4D flow MRI) [33, 40]. The typical fitting targets for 3D cardiac modeling are motion fields or PV relationships. Chen et al. [40] inversely estimated the passive material parameters of the Holzapfel–Ogden law from the in vivo LV end-diastolic (ED) volume and myocardial strain data using the multistep optimization method (i.e. the *lsqnonlin* function in MATLAB, The MathWorks, Inc.) [112]. Caforio et al. [33] used the model function-based fitting method [110] to match the passive biomechanical material properties of the Guccione law to an empirical Klotz ED PV relationship estimated from a single measurement [114] and determine the undeformed reference configuration simultaneously. In most studies [33, 48, 51, 110], the stress-free reference configuration of the heart, which is crucial for accurate modeling of biomechanical diastolic function, was established based on loaded in vivo images by applying the unloading and reinflation method through fixed-point iterative techniques [48, 115, 116].

With regards to the valve model, geometrical parameters such as the orifice area have been obtained using either CTA or transthoracic Doppler echocardiography techniques [37]. On the other hand, the EOA of the AV, calculated as the ratio between the stroke volume (SV) and velocity–time-integral of the peak aortic flow velocity, has been derived based on transthoracic Doppler echocardiography or from cardiovascular magnetic resonance images [42].

Systemic vascular resistance (SVR) and arterial compliance parameters in a simplified 3-element WK model are usually adjusted to reproduce the measured systolic, diastolic, and mean arterial pressure at a measured average flow rate. Sadeghi et al. [49] utilized the Simulink Design Optimization toolbox in MATLAB (The MathWorks, Inc) to tailor the lumped parameter systemic circulatory model response through two sequential automatic steps with tolerances of 10^–6^. On the other hand, Veress et al. [66] employed the SENSOP optimizer [111] to adapt the circulatory model (i.e. the systemic resistance and capacitance parameters) to the pressure/volumes generated by the LV FE model. On the other hand, parameterization of a 1D arterial model is more cumbersome due to the topological complexity of the arterial tree. Reymond et al. [117] has proposed a systematic approach to personalize a 1D arterial model based on subject-specific measurements that has been utilized in several studies [35, 36, 39, 41]. Firstly, the geometrical measurements, including diameter, area and length of the individual arterial segments are obtained using MR angiography. Temporal waveforms of the volume flow rate at several SA locations are then derived from time−velocity waveform and cross-sectional area information acquired using 2D-gated phase-contrast MRI as well as B-mode and color-coded duplex flow imaging. Pressure waveforms are measured at superficial arteries using applanation tonometry, calibrated using brachial sphygmomanometer measurements [117]. Lastly, arterial stiffness is optimized by minimizing the difference between the simulated and measured PWV, which is derived from the pressure waveforms at the carotid and femoral arteries. Local arterial distensibility is taken to be a function of the transmural pressure and lumen diameter.

## Model coupling and computational resources

Numerous coupling approaches have been implemented to integrate different cardiovascular components in a VA model, which vary from single- to multidimensional compartments. In the simplest models, such as those which modeled all compartments using the lumped parameter representation, coupling is achieved by ensuring that conservation of mass (flow rate) is satisfied [38]. In a 1D arterial tree model, coupling between different arterial segments at the bifurcations is accomplished using a ‘ghost-point’ method solved iteratively with the Newton–Raphson technique [57, 58].

The most common coupling method in a VA model works by imposing continuity of hemodynamic variables (i.e. flow rate and pressure) at the coupling interfaces. Normally, a time-stepping iterative algorithm involving three steps, i.e. (i) initialization, (ii) iteration setup, and (iii) convergence assessment, is applied. During the initialization phase, a hemodynamic variable (e.g. pressure) is prescribed at the coupling interface based on empirical data. During the iteration phase, the state variable (e.g. flow rate) is computed by running the model (e.g. the LV) using the prescribed BC. The computed state variable (e.g. flow rate) is subsequently used as BC for the adjacent model (e.g. the arterial network). Upon solving the adjacent model, the hemodynamic variable (e.g. pressure) at the coupling interface will be updated to be used for the next time step. During the convergence assessment phase, a synthesized iterative error for the hemodynamic variable (i.e. flow rate or pressure) at the coupling interface is calculated. Liang et al. [56] applied the above technique to integrate the structured-tree models of the distal arteries/arterioles with the upstream 1D model of the large arteries and downstream 0D model of the capillaries. Similar technique is applied by Chen et al. [40] to couple a 3D FSI LV model with a 1D SA model during the systolic ejection phase when the AV is open.

On the other hand, a few studies have coupled a 1D arterial network to a lumped parameter (0D) description of the remaining circulatory system and integrated the solutions at the 1D−0D interface using the time−marching iterative method [45, 56–58]. The 1D blood flow equation is transformed into the characteristic variables of a hyperbolic system (also known as Riemann invariants), as represented by W_1_ and W_2_. W_1_ and W_2_ denote the forward and backward traveling wave leaving the domain through the distal and proximal nodes, respectively. The resting conditions (i.e. initial vessel area and zero flow rate) are typically chosen as the reference state to derive the characteristic invariants expression in relation to the flow velocity, cross-sectional area, and flow rate. At each time step, the process begins with extrapolating the Riemann invariants (e.g. W_1_) in the 1D model to estimate flow rate at the 0D−1D interface (e.g. peripheral arterial distal interface), which acts as a BC for computing pressure in the 0D model. Based on the calculated pressure, the cross-sectional area of the vessel is derived and then fed back to the 1D model to calculate the new flow rate based on the derived Riemann invariants (e.g. W_2_) and the current flow velocity. The residual error is computed based on the difference between the estimated and the new flow rates.

To couple a four-chamber heart model with a reduced-order vascular model, Caforio et al. [33] impose a coupling condition which requires that the volume change in each heart cavity is balanced with that in the attached vascular system. By reinterpreting the LV cavity pressure as a Lagrange multiplier, a volumetric constraint was enforced to couple the 0D circulation model with the 3D EM model. This resulted in a saddle-point problem involving the displacement and LV cavity pressure variables to be solved via the Schur complement reduction approach [33, 48]. Caforio et al. [33] stated that the volume of each cardiac cavity equals an initial volume and does not change during the isovolumetric phase. Similarly, Regazzoni et al. [48] adopted the volume−consistency coupling condition for their coupled 3D (LV EM)-0D (circulation) model, but solved their model in both a segregated and staggered manner instead of the more common monolithic approach, discretising the 0D and 3D models simultaneously as a coupled system. The fully segregated approach enables the use of different discretization in space and time to approximate variables associated with the different component models. Although the majority of VA models utilize direct coupling, it is computationally expensive to couple between two distinct dimensions in a multidimensional model, for example, linking a 3D FE method LV mechanical model to a 0D circulatory system based on two different software platforms. Veress et al. [66] have instead used a weak coupling method, where the transfer of information between the 3D FE method LV and the 0D circulatory model is unidirectional instead of bidirectional.

Furthermore, several studies [34, 40, 45] had been conducted to analyze the outcome difference between isolated SC or cardiac model and fully coupled heart-circulation model. The results showed that the isolated SC model overrates peak pressure [34] and flow rate [40] and underestimates the reflections [45] in pathological situations when compared to the VA coupled model. This might be due to the imposed inflow not adapting to the arterial conditions. The sensitivity to the variation of arterial stiffness of the model using an uncoupled arterial model (prescribed inlet BC) is significantly lower than in the coupled model (coupled 0D heart model) in middle-aged individuals [45]. Compared to the uncoupled cardiac model (0D circulation instead of 1D), only the aortic pressure curve obtained with the fully coupled model shows a dicrotic notch, which is characterized by a small downward deflection in the pressure contour on the downstroke of the aortic pressure waveform following the systolic peak, that marks the closure of the AV [34]. These findings highlighted the importance of VA coupling and hence modeling and coupling both cardiac and circulatory system should be considered.

The required computational resources to run a VA model can vary significantly, depending on the complexity of the simulation, the scale of the problem, and the precision required. Numerical simulations were run by employing life^x^ [118, 119] in parallel on either the High Performance Computing (HPC) resource (48 Intel Xeon ES-2640 CPUs) or the GALILEO supercomputer (252 cores distributed across 7 nodes with 36 Intel Xeon E5-2697 v4 2.30 GHz CPUs). Apart from adopting more efficient computational methods, another approach to save computational resources is to reduce model complexity. Syomin et al. [65] modeled their LV as 2D-axisymmetric geometry, as opposed to of 3D, and reported that their 2D(LV)-0D(circulation) model took only 5 min to compute a 1 s evolution of the cardiovascular variables using a workstation with two 12-core processors. In comparison, the 3D(LV-FSI)-1D(arterial) model developed by Chen et al. [40] took approximately 168 h to complete one cardiac cycle on a local Linux workstation with eight Intel® Xeon® CPU cores (2.65 GHz) and 32 GB RAM. With the availability of cutting-edge computational resources, computing constraints associated with complex multidimensional models could be mitigated.

## Model applications

Computational models of VA coupling have been used to investigate aging [34, 36, 57, 59], HTN, ventricular diseases (e.g. LVH, LVR, and LV stiffening) [36, 52], valvular diseases (e.g. AS, AV regurgitation (AR), MV stenosis (MS) and MV regurgitation (MR)) [42, 47, 50, 51, 61, 65] as well as vascular diseases (e.g. arterial stiffening, atherosclerosis, arteriosclerosis, COA, aneurysm and rarefaction) [33, 38, 41, 45, 46, 56, 66], as indicated in Table 2. In some cases, a combination of several complications, such as systemic HTN, AS and LVH were considered in the same study to assess their interactions [44, 60].

### Aging and hypertension

Computational models have been used to investigate mechanisms leading to systemic HTN in aging and its hemodynamic consequences. In most studies [39, 41, 45, 56, 58], 1D model has been used to simulate arterial system due to its accuracy in reproducing pulse wave propagation and reflection phenomena which is an important determinant in HTN. Aging-induced aortic stiffening and remodeling exhibit a compensatory effect on aortic systolic BP and PP as well as PP amplification [41]. Aortic dilatation can partly counteract the increased aortic systolic BP and PP induced by arterial stiffening due to premature wave reflection, which leads to systemic HTN [57]. On the other hand, aortic stiffening tends to reduce reflection coefficients at vessel bifurcations, thus enhancing the protective wave-trapping mechanism on the reflected pressure waves [41]. This can reduce PP amplification associated with dilatation of the aorta, which subsequently reduces the detrimental risk impacts on organs such as the kidneys.

Computational models of VA coupling have also been used to investigate mechanisms leading to alterations in aortic pressure waveforms in diseased states, which is an important trigger for systemic HTN and LVR [34, 39]. While reduced aortic compliance associated with aging has been reported to change the aortic pressure waveform phenotype from Type C (i.e. peak systolic pressure precedes the inflection point) to Type A (i.e. peak systolic pressure occurs after the shoulder) due to early wave reflection, recent studies have found Type C pressure phenotype in old age HTN [120]. Using simulation studies, Pagoulatou et al. [36] showed that an increase in LV contractile strength caused by LVR (adaptation mechanism to an increase in the systemic afterload) alters the shape of the forward pressure wave, making it steeper and reaching its peak early. This alters the central hemodynamics, restoring the Type C pressure phenotypes and increasing systolic pressure augmentation. As a result, indices based on the aortic pressure waveform, such as the widely applied surrogate of wave reflection, AIx (defined as the ratio of the augmentation of systolic BP to PP), cannot be assumed to reflect only arterial properties, but are instead dependent on both vascular and cardiac properties such as LV contractile properties [35, 36, 53]. Besides, both heart and arterial system play a major role in contributing to BP changes in HTN and aging [59, 63].

VA coupling models have also been applied to study the associations between cardiovascular properties and hemodynamic parameters and elucidate the differential effect of various antihypertensive drugs on hemodynamics. Using a computational model with single-factor sensitivity analysis, Liang et al. [56] found that central arterial wall stiffness, heart period, and arteriolar radius are significant determinants of arterial BPs. Vasodilators have comparable efficacy in reducing central aortic stiffness that resulted in more significant decrease in aortic PP and a significant rise in aortic-to-brachial PP amplification ratio. This could be explained by the positive effect of vasodilators on the relaxation and dilatation of distal resistance vessels. On the contrary, beta-blockers yield a reduction in HR with opposite effect of that provided by a reduction in central aortic stiffness.

### Ventricular function

With an increase in systemic afterload, the LV undergoes adaptation in the form of concentric hypertrophy, in which LV wall thickness increases to normalize peak systolic wall stress, whereas SV is preserved via a surge in preload (Frank−Starling mechanism) and a rise in LV contractile strength. This cardiac remodeling process further contributes to increased LV and aortic peak systolic pressure. The coupled LV systolic and arterial stiffening helps to preserve LV mechanical performance at the expense of further elevation of LV and aortic pressures. In addition, detailed CFD analysis has shown a much larger energy loss in patients with LVH (compared to control and non-LVH groups), which is caused by a disturbed (turbulent) flow field with disordered/irregular vortices in the LV [52].

VA coupling models have been used to assess the sensitivity of myocardial function indices to cardiovascular system variations. For example, with the use of complete 0D CL cardiovascular model, Inuzuka et al. [55] analyzed the effect of HR, LV contractility, diastolic stiffness or relaxation time, arterial compliance and SVR on myocardial performance index (MPI), derived from Doppler measurements, serves as an index for overall ventricular function by estimating the combined systolic and diastolic LV performance. Their simulation studies showed that MPI acts contrarily to diastolic dysfunction resulting from impaired LV relaxation and LV diastolic stiffening, as well as responding differently to static (SVR) and pulsatile (arterial compliance) afterload. On the other hand, the Ea/Ees ratio has commonly been used to assess the balance between the load imposed by the arteries and the contractile properties of the LV. Although the Ea/Ees ratio is a widely accepted index for charactering left VA coupling, it has been shown to depend on aortic leak severity, and therefore could not reliably reflect LV−arterial coupling in patients with AR [61].

### Valvular and arterial diseases

Computational models of VA coupling have also been developed to investigate the effect of valvular diseases on both the heart and arterial system. Liang et al. [58] observed from their simulation studies that AS induces early wave reflection and leads to prolonged LV ejection, delayed peak transvalvular flow, as well as increased LV systolic pressure, which may in turn trigger hypertrophic remodeling and hamper myocardium relaxation. In order to accurately capture the large transvalvular pressure gradient associated with severe AS, the flow coefficient parameter used in the valve models should be allowed to vary depending on the opening diameter of the vena contracta (rather than setting it as a constant), as blood flow Reynolds number can change significantly over the ejection period and over the various degrees of stenosis [47, 65]. In another study [49], mixed valvular diseases, such as aortic regurgitation and mitral regurgitation, have been shown to alter velocity magnitude downstream of the COA, creating turbulent flow and increasing the pressure gradient across the coarctation. Consequently, it has been suggested that the severity of valvular diseases should be considered in the evaluation of risks and selection of treatment approaches in patients with COA.

VA coupling models have also been applied to assess the sensitivity of biomechanical markers to variations in the cardiovascular system, and to identify new biomechanical markers which could better diagnose and assess disease progression in patients with valvular diseases. To date, transvalvular pressure gradient, EOA and ejection blood jet velocity have been used in routine clinical practice to identify the presence of AS [121]. However, these diagnosis criteria are unreliable in the presence of LV dysfunction coupled with impaired SA compliance. For example, in a low flow, low gradient AS scenario, an elevated transvalvular pressure gradient is lacking due to ventricular dysfunction [122]. These patients have reduced LV function coupled with eccentric hypertrophy (spherical-shaped LV) from pathological remodeling. Using a biomechanical model, Wineski et al. [50] observed a reduction in systolic myocardial stress with more uniform stress distribution throughout the LV in these patients. On the other hand, echocardiography measures of aortic regurgitation severity, such as regurgitant volume, regurgitant fraction and pressure half time, as well as its hemodynamic consequences such as mean left atrial pressure, are influenced by LV diastolic stiffness and aortic wall stiffness [60]. These findings highlight the importance of taking into account both cardiac and arterial tissue properties while assessing the severity of valvular diseases using existing clinical assessment methods.

In order to more reliably assess hemodynamic load imposed on the LV, the Zva index has been proposed, quantifying both valvular and arterial loads on the LV [123]. Zva is closely associated with peak systolic WSS and aortic wall stress in patients with ATAA, proving to be a robust predictor of LV dysfunctions and clinical outcomes in asymptomatic AS patients [37]. Another study [42] has proposed the normalized LV SW to evaluate overall hemodynamic load imposed on the LV. While Zva is flow-dependent and offers an estimate of LV load, the normalized LV SW is not flow-dependent and determines the actual mechanical load imposed on the LV which is more suitable to be used on low flow, low gradient AS patients.

## Future perspectives

**Fig. 6:**
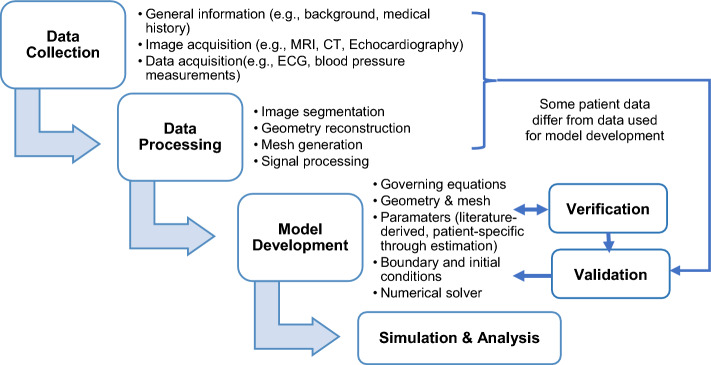
Patient-specific computational model framework involving data collection, processing, model development, and lastly simulation and analysis

Promising future research directions in cardiovascular modeling are the development of patient-specific fully coupled cardiovascular modeling frameworks (Fig. 6) (i.e. four-chambered heart, valves, pulmonary and systemic circulatory systems that include arterial and venous systems) in concomitant pathological conditions which can provide clinically-relevant insights for management of diseases for individual patients and improve clinical decision making and patient outcomes. CL model structure is preferable due to it can account for LV preload−afterload interaction. To date, fully integrated multiscale 3D whole heart EM models coupled with 0D CL circulatory models have emerged, which highlighted the significance of considering atrial contraction and assessing the impact of local cardiac tissue changes on the entire system [82, 124]. Additionally, Bucelli et al. [89] has introduced a 3D model of the human heart which included detailed descriptions of the electrophysiology, active and passive mechanics as well as fluid dynamics, coupled with reduced models for valves and circulation. Despite their detailed and accurate representation of the fully coupled cardiovascular system, these models are computationally complex and expensive, posing challenges in optimizing patient-specific hemodynamic parameters due to the limited availability of noninvasive measurements, especially within a CL circulation framework. To overcome the challenge for model parameterization, uncertainty quantification and sensitivity analyses [125–127] should be performed to determine and prioritize the most influential parameters through clinical measurements, with remaining parameters fixed or derived from the literature. Personalized parameters can be estimated using data assimilation and optimization algorithms with robust inverse problem strategies (e.g. unscented Kalman filter [128, 129], adjoint-based optimization for cardiac mechanics [130]) to better fit available measurements to improve model accuracy [131]. Reduced-order modeling should be considered by simplifying the system or geometry to reduce computational complexity. Like 1D arterial models, the parameter set can be reduced by decreasing the number of arterial segments involved via lumping peripheral branches into WK-type models which can still reproduce the desired features of BP and flow waveforms [132]. Clinical measurements (i.e. body mass index, PWV) can be utilized to adjust distal properties and vessel geometry using allometric scales and global descriptors associated with the network's overall geometry and properties [117].

Furthermore, future studies should centre on the development of more detailed and realistic modeling of valves, right heart, and the pulmonary and venous circulation to consider their interactions in the cardiovascular system. The effect of these components in the study of VA coupling are uncertain, in which models typically focus on the left heart and systemic circulation whilst the pulmonary circulation and right ventricle (RV) are not emphasized [103, 133], even though the latter are important in determining overall performance of the cardiovascular system, including LV and aortic mechanics [134]. This lack of emphasis may be due to the challenges in personalizing these models, namely that right side of the heart is hard to segment and variables of the right heart and pulmonary circulation (i.e. pulmonary arterial pressure) require invasive catheter measurements [69, 135] which are not easily accessible. Alternatively, the use of noninvasive echocardiography in assessing pulmonary arterial pressure [136] can be considered for patient-specific modeling. Besides, the effect of cardiac valves is ignored or not emphasized in most coupled cardiovascular models. Venous return, which affects cardiac preload, should also be studied more precisely to understand the effect of the circulation on cardiac function.

Besides, another issue is the computational limitation that each model requires an extensive development time to formulate appropriate mathematical descriptions of the underlying physiology. The use of patient-specific and high resolution in silico models in clinical practice is still not currently feasible due to long computation time and comprehensive guidelines required. This has led to a growing interest in machine learning and deep learning methods [137] to reduce computational cost with improved outcomes, rather than applying a complex mathematical model of the many physiological systems present. Instead of complete modeling, machine learning can also be applied for automated segmentation of medical images to reconstruct the 3D geometry [138]. Although machine learning techniques have been recently deployed to learn the relationships among different cardiovascular parameters, it is recognized that its “black box” nature poses additional challenges in the context of verification and validation [139]. To deploy deep learning for cardiovascular care, challenges in obtaining substantial labeled data, enhancing interpretability and robustness, and creating standardized methodologies for validation and testing need be solved. To accurately quantify the effects of patient variability on physiology, pathophysiology, and treatments, as well as to make predictions using deep learning algorithms, it will be crucial to develop and utilize virtual patient cohorts [140, 141].

Future studies should also focus on assessing the efficiency of existing prognostic indicators of cardiovascular function. To ensure efficiency of the prognostic indicators, the sensitivity of cardiac and vascular function markers to variations in cardiovascular properties should be evaluated to enable clinicians and researchers to more clearly interpret clinical/experimental results related to these markers in aging, HTN, and concomitant disease. Further investigation is necessary to develop indices that accurately reflect specific conditions and enhance the evaluation of disease severity and its hemodynamic implications. New indices that can perform a comprehensive and precise assessment of a patient's true hemodynamic and clinical condition should be introduced.

However, most studies [56, 64] only focused on isolated changes of parameters which might not be the actual case. To account for variations in hemodynamic conditions among patients and the interrelation of cardiovascular factors in vivo, encompassing diverse short-term regulatory and long-term adaptive mechanisms, it is important to investigate the collective impact of parameters.

The limitation of this review is that only left VA coupled models, the influence between cardiovascular components, and disease progression are discussed. Future studies can investigate literature pertaining to right VA coupled models and the effect of interventions. The limitations of evidence are the lack of explanation of fundamental information due to the same previous model being applied. The limitations of review processes are that too many specific search terms were applied. However, it should be noted that the use of more generic search terms might result in too much nonspecific literature. Another limitation is that only three databases were used to obtain relevant papers. Furthermore, additional searches via other methods employed may potentially lead to bias in this review.

## Conclusion

The application of computational models for examining impaired left VA coupling in aging and disease have been reviewed and discussed. The notion of VA coupling provides important insights in cardiovascular system analysis. It is crucial to evaluate the heart and vessels as an interconnected system instead of isolated structures. The choices of dimensionality in multicomponent models might be insufficient to replicate significant features of pathological change during aging, HTN, and complex ventricular−valvular−vascular disease. Future research directions should involve the development of patient-specific fully coupled models of the cardiovascular system by incorporating models with appropriate dimensionality that the choice should depend on the level of detail required in the simulation and the available computational resources. The implementation of machine learning techniques can be considered for model prediction or data acquisition and processing. The sensitivity of cardiac and vascular function markers to changes in cardiovascular system properties should be analyzed to determine the efficacy of these indicators and allow clear interpretation of clinical results.

## Systematic review methods

A systematic review procedure was implemented according to the Preferred Reporting Items for Systematic Review and Meta-Analyses (PRISMA) [142] guidelines. A comprehensive literature search was undertaken for relevant articles dated up until 14th of July 2022 using electronic databases such as Web of Science, Scopus, and PubMed, as well as other search methods including websites and citation searching. Keywords comprised “ventricular−arterial”, “computational model”, and their respective synonyms and related terms with the use of truncations and the Boolean operators AND and OR were applied within all fields in the databases. Details of the search term combinations are provided in Table 1. To limit the number of articles in the preliminary stage, available database filters were employed with several conditions (Additional file 1: Table S1). Journal articles published in English were included, and focus areas were specified to be engineering, cardiovascular system and cardiology, mathematics, physiology, and computer science. A publication timeframe from 2000 to 2022 was adopted to ensure that recent significant and advanced modeling methods were retrieved.

**Table 1:** Details of search terms used in the databases

Key concept	*VA Coupling*		*Computational model*
Search Terms	“ventric*-valv*-vascula*" OR "ventric*-valv*-arter*” OR “ventric*-vascula*" OR "ventric*-arter*” OR "cardiovascular system" OR "arter*-ventric*" OR “ventric*-aort*” OR “heart-vessel” OR "heart-arter*" OR "heart-vascular*" OR “cardi*-arter*” OR “cardi*-arterial”	AND	"3D-0D" OR "3D-1D" OR "0D-1D" OR "mathematical model*" OR "computational fluid dynamics" OR "fluid–structure interaction" OR "finite-element" OR "interact* model*" OR "numerical model*" OR "lumped parameter model*" OR "comput* model*" OR "multi-scale model*" OR "multi-physics model*" OR "in silico"

Database search results were subsequently imported into EndNote 20 (Clarivate, USA) where duplicates were found and removed. Eligibility criteria for this review were (1) usage of a computational model of the cardiovascular system, (2) use of human cardiovascular data, particularly in adults, (3) coupling between the LV and the aorta, (4) it must involve the systemic circulation, (5) it must involve age-induced complications or other left ventricular–valvular–vascular related disease without interventions, and (6) modeling approaches are well defined (Additional file 1: Table S2). The screening of titles, keywords, and abstracts of the articles was initially undertaken to eliminate irrelevant studies. Full papers were then sought for retrieval, reviewed for relevance, and screened for quality to ensure articles satisfied the above eligibility criteria.

**Fig. 7:**
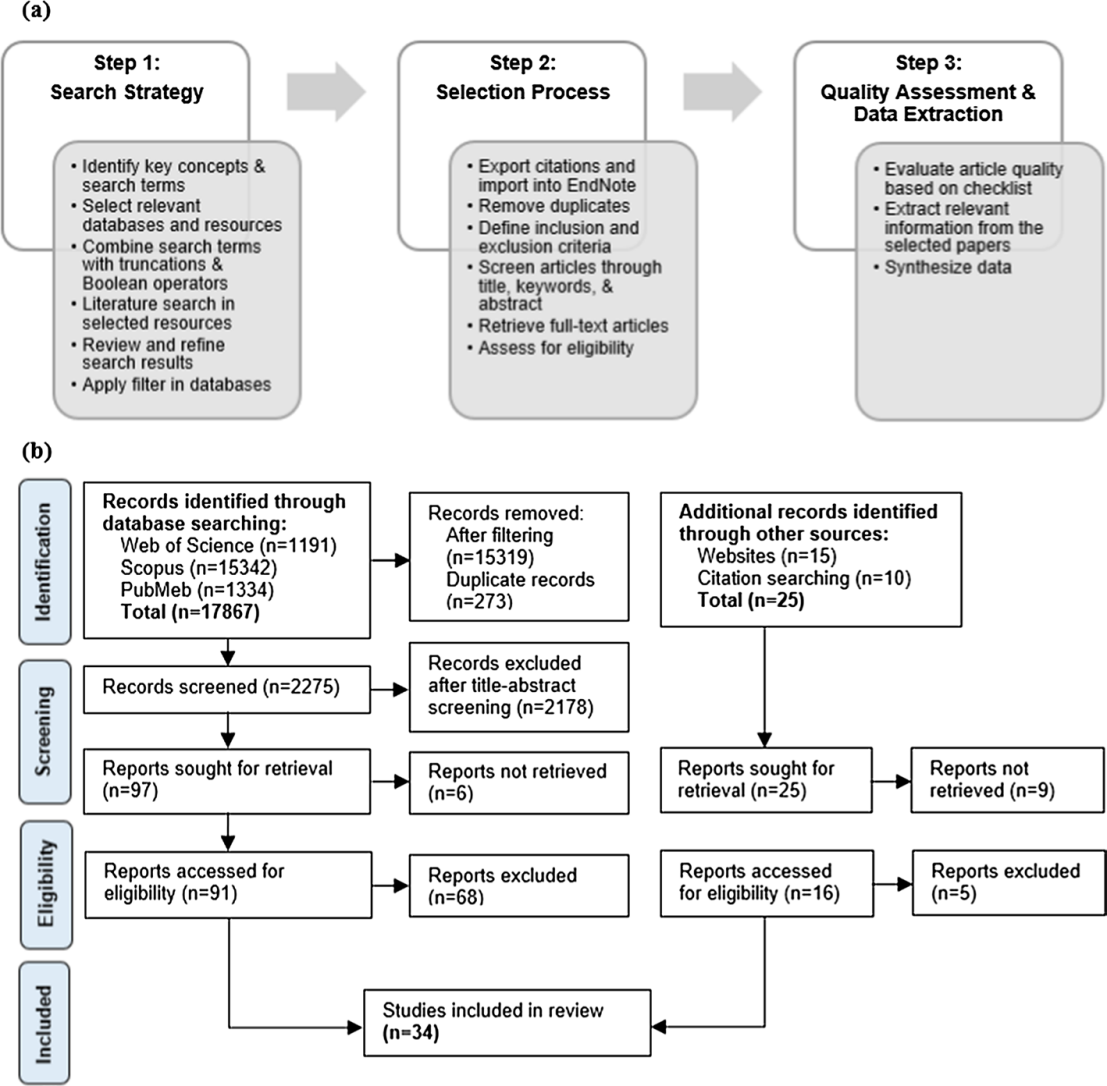
Systematic review approach: **a** Overview of methodology; **b** PRISMA flow diagram of study selection process adapted from the PRISMA 2020 statement: an updated guideline for reporting systematic reviews [142]

Critical appraisal of the selected articles was conducted to ensure their usefulness towards this review. To avoid bias, a grading system was prepared to assess the quality of the papers which included a set of questions relevant to a systematic review of computational modeling [143–146], including questions pertaining to study objective, data source, modeling technique, model parameterization, uncertainty assessment, simulation, model validation, results, key findings, limitations, and conclusions (Additional file 1: Table S3). The assessments were completed separately by two reviewers (CCAD, LE) (Additional file 1: Table S4), and selected articles were organized in Microsoft Excel (Microsoft Inc, USA) spreadsheet. Information such as authors, publication year, model application, modeling technique, and key findings of the study were extracted from each study for further interpretation. Figure 7 illustrates the methodology and selection process. The general overview of review findings is summarized, including authors, publication year, model development (model structure, parameterization, and validation), and model application (applications and key findings of each study) (Table 2). Detailed information about model development (Additional file 1: Table S5) and model application (Additional file 1: Table S6) were provided in supplementary material. The studies were sorted based on year of publication to highlight the latest research and modeling techniques.

**Table 2:** Summary of included studies

Authors	Application	Model structure				Parameterization	Validation	Key findings
		Type	Heart	Valve	Circulation			
Caforio et al. (2022) [33]	Aortic stiffening, COA, aging	OL	3D EM LV (MRI)	0D valve dynamics [93]	1D upper thoracic aorta; 116 SA + 3-element WK	Patient-specific data (MRI and invasive BP)	MRI clinical data under baseline conditions	Arterial stiffening increased ES volume with ED volume unaltered, resulting in a drop in SV. Increased aortic stiffness due to stenosis or aging with increased SVR caused increased peak pressure, changes in pressure profile, and a drop in SV due to a rise in ES volume
Laubscher et al. (2022) [47]	AS	CL	0D TVE	0D (M1: diode; M2: pressure loss [147]; M3: valve pressure loss and motion)	0D systemic and pulmonary circulation	Population-averaged data (literature-derived)	Typical human physiological hemodynamic parameters	The proposed M3 valve model predicted higher pressure drops at severe AS than the M1 and M2 models from literature
Regazzoni et al. (2022) [48]	HTN	CL	3D EM LV + 0D TVE LA, RA, RV	0D nonideal diode	0D systemic and pulmonary circulation	Generic data (literature-derived)	Not mentioned	Atrial contractility affected preload and SV positively. Increased arterial resistance raised AV opening pressure and maximal LV pressure (hypertensive effect). Increased myocardial contractility raised maximal LV pressure and SV
Wisneski et al. (2022) [50]	low flow low gradient AS	CL	3D FE LV (CT)	0D MV and AV	0D systemic and pulmonary circulation	Patient-specific data (echo and catheterization)	Patient clinical parameters	Contrary to idealized LV geometry and normal ventricular function, a patient-specific LV model with low flow, low gradient AS revealed a quantifiable reduction in LV stress
Zuo et al. (2022) [52]	Hypertensive myocardial hypertrophy	CL	3D CFD LV (as BC); 0D TVE LV, RV, LA	0D (diode and resistor)	0D resistance−inductance−capacitance systemic and pulmonary circulation	Generic data (literature-derived [148])	Echo measurements and MRI data on MV and AV	Myocardial hypertrophy due to HTN affected flow domain, leading to abnormal vortex distribution, higher energy loss, lower blood flow velocity, and low cardiac EF. In comparison, the energy loss and velocity distribution of HTN normal LV group were like the normal LV control group, but with slightly higher characteristic parameter values
Sadeghi et al. (2022) [49]	COA; mixed valvular diseases	CL	0D TVE LA, LV	0D MV and AV (net pressure gradient)	3D CFD thoracic aorta (CT) + 0D COA, systemic and pulmonary circulation	Patient-specific data (Doppler echo and sphygmomanometer)	Doppler echo, cardiac catheterization and 4D flow MRI data	Coexistent AR and MR with COA changed downstream velocity and created turbulence, leading to disease progression at the COA region
Manganotti et al. (2021) [34]	Aging	OL	0D (Hill-Maxwell)	0D (diode and resistor)	1D upper thoracic aorta + 3-element WK	Generic data (literature-derived [149])	Not mentioned	Aging was associated with higher systolic peak, lower diastolic BP, and a slightly increased wave propagation speed (anticipated dicrotic notch). Coupled model yielded more physiological pressure curve (trend towards merging of pressure systolic peak and dicrotic peak). Uncoupled aortic model in aging case showed nonphysiological double reflection
Pagoulatou et al. (2021) [35]	LVR, HTN	OL	0D TVE LV	0D	1D 103 SA + 3-element WK	Generic data (literature-derived)	Applanation tonometry, phase-contrast MRI and echo data	Reducing proximal aortic compliance acutely increased aortic systolic BP and PP, leading to HTN, which is partially alleviated after LV remodeling. Banding increased the forward wave amplitude, which further increases PP, and LV remodeling caused the forward pressure wave to alter its shape, resulting in a distinct upstroke and an earlier peak. The primary factor driving the transformation of the pressure waveform from an old to a young phenotype was identified as LV remodeling
Pagoulatou et al. (2021) [36]	Cardiac inotropy	OL	0D TVE LV	0D	1D 103 SA + 3-element WK	Generic data (literature-derived)	Applanation tonometry, phase-contrast MRI and echo data	AIx, based on the pressure waveform, did not exclusively reflect arterial properties, as cardiac contractility also plays a crucial role in determining central AIx
Cosentino et al. (2020) [37]	ATAA, AS	OL	0D	–	3D FSI aorta (CTA) + 3-element WK	Patient-specific data (clinical and echo data)	Echo data on AV	LV work increased with AS severity, with post-stenotic variables (including WSS) markedly increasing, particularly for severe AS models. Higher WSS and maximum principal stress of ATAA wall were associated with more severe LV dysfunction indicated by Zva
Wisneski et al. (2020) [51]	AS	CL	3D FE	–	0D systemic and pulmonary circulation	Generic data (literature-derived)	Tagged MRI data	Global LV peak systolic myofiber stress increased progressively with AS severity, while ED stress remained relatively constant across all conditions
Heusinkveld et al. (2019) [53]	Cardiac inotropy, vascular aging, cardiac and vascular tissue changes	CL	0D (modified Hill)	–	1D TL arterial and venous tree + 0D peripheral circulation	Generic data (literature-derived [150])	Applanation tonometry data	Both LV contraction velocity and increased arterial stiffness affected AIx. However, a rise in AIx did not necessarily correspond to a rise in LV SW. Wave reflection magnitude, determined by considering both pressure and flow, was also a factor in determining LV SW
Syomin et al. (2019) [65]	AS, AR, MS, MR	CL	2D axisymmetric FE LV + 0D atria and RV	0D (diode, resistor, inductor, capacitor)	0D systemic and pulmonary circulation	Generic data (typical values)	Published clinical data	AS: Reduced AV maximal orifice area resulted in a rise in the mean and maximal pressure difference between the LV and aorta, along with a decrease in LV ED and ES volumes, SV, and EF MS: LV ED volume and SV decreased significantly at a constant blood volume AR and MR: Regurgitant volume and fraction increased with the maximal orifice area of valve
Gul et al. (2019) [38]	Aortic stenosis and aneurysm	OL	0D TVE LV	0D MV and AV (diode)	0D 122 systemic circulation	Generic population data (literature-derived)	Not mentioned	In the presence of aortic stenoses (aneurysms), node 34 (33) had a greater impact on pressure and flow than node 33 (34). Sensitivity of pressure and flow in the systemic circulation to stenoses and aneurysms increased with higher HRs
Shavik et al. (2018) [64]	Aortic remodeling (wall thickening and stiffening), LV stiffening	CL	3D FE half prolate ellipsoid LV	0D MV and AV	3D FE idealized aorta + 0D systemic circulation	Generic data (literature-derived)	Population-averaged in vivo data from different literature	Increasing aorta wall thickness caused a lower LV EF, higher peak LV systolic BP, and leftward shift in the aorta pressure-diameter relationship with smaller diameter at ED and ES. Elevated collagen mass increased peak systolic BP but reduced LV EF. Decreasing LV contractility and increasing passive stiffness lowered LV EF, aortic systolic BP, PP, and peak stress
Liang et al. (2018) [56]	HTN, arterial stiffening	CL	0D TVE	–	1D 55 SA + ST + 0D pulmonary circulation, capillaries and veins	Population-averaged data (literature-derived)	Normal human data under physiological condition	BP and flow pulsatility indices in both large arteries and microcirculation were mainly determined by heart period, arteriolar radius, and central arterial stiffness. To fully account for the pressure-lowering effects in the aorta, central arterial stiffness must be reduced simultaneously with the structural normalization of distal vessels
Pagoulatou et al. (2017) [39]	Aging	OL	0D TVE LV	0D	1D 103 SA + 3-element WK	Population-averaged data (literature-derived)	Published data from large-scale clinical studies [151, 152]	The forward wave was the main cause of central and peripheral systolic BP and PP increase with age due to a stiffening proximal aorta that augmented it. AIx steeply increased in young adults but declined after 60 years
Maksuti et al. (2016) [59]	Aging	CL	0D TVE LV	0D (diode)	0D 4-element WK SA	Generic data	Population data (Framingham Heart Study)	Arterial and cardiac factors both contributed to age-related changes in BP. Arterial changes led to a rise in systolic BP, which triggered cardiac remodeling, further increasing systolic BP and mitigating the decrease in diastolic BP
Chen et al. (2016) [40]	Arterial stiffening, rarefaction, LVH, inotropy	OL	3D FSI (MRI)	3D passive AV	1D 24 SA + ST	Subject-specific and generic data (MRI, literature-derived)	Published experimental data (healthy subjects)	Arterial stiffening and rarefaction led to higher BP, LV active tension, but decreased SV. LV stiffening caused severely impaired pump function, reducing active tension, SV, and BP. Elevated contractility could maintain a higher SV but raised circulation pressure. Isolated systemic circulation model overestimated peak pressure (up to 7%) and flow rate (up to 20%) compared to a coupled model
Inuzuka et al. (2016) [55]	HF progression with chronic mitral regurgitation	CL	0D modified TVE	–	0D systemic and pulmonary circulation (modified 3-element WK)	Generic data (literature-derived [153])	Echo data	Increase in HR decreased EF and increased MPI. Ees reduction also decreased EF and increased MPI. Volume overload and ventricular stiffening decreased MPI. Higher SVR increased afterload, leading to decreased EF and increased MPI, while afterload decrease due to reduced arterial compliance decreased both. These MPI characteristics led to paradoxical MPI improvement during chronic HF disease progression in a simulation of MR
Palau-Caballero et al. (2016) [60]	AR, LV and aortic stiffness	CL	0D	0D	0D aorta, pulmonary and peripheral circulation	Generic data (literature-derived)	Echo data across AV from AR patients	AR severity scores (regurgitant EOA, regurgitant fraction, pressure half time) poorly reflected mean left atrial pressure when variations in tissue properties (LV and/or aortic stiffness) were present
Guala et al. (2015) [41]	Aging, aortic stiffening and remodeling	OL	0D TVE LV	0D AV dynamics	1D SA + 3-element WK	Generic data (literature-derived)	Arterial tonometry	Aging-induced aortic stiffening amplified the first pressure pulsed at the VA interface, while remodeling suppressed it. Though stiffening tended to decline reflection coefficients at network bifurcations, the substantial growth induced by remodeling prevailed, raising the overall amount of reflection. Aortic remodeling undermined the protective wave-trapping mechanism on reflected pressure waves, whereas stiffening improved it. Both aortic stiffening and remodeling had a compensatory effect on PP amplification, with the former reducing it, and the latter increasing it. Together, they helped restrain the LV work growth associated with aging
Keshavarz-Motamed et al. (2014) [42]	AS	OL	0D TVE	0D AV (diode, variable resistor, inductor)	0D systemic circulation	Population-averaged data (transthoracic Doppler echo and MRI)	MRI data (healthy subject and AS patient)	The proposed normalized LV SW correlated well with Zva, a validated index of global hemodynamic load, and was less flow-dependent than Zva
Veress et al. (2013) [66]	HTN	CL	3D FE LV (as BC); 0D TVE LA, LV	–	0D WK systemic circulation	Generic data (literature-derived)	Not mentioned	Mild and moderate HTN caused an increase in cardiac output and SV compared to normotension. Even mild HTN could significantly increase total LV wall stress. A moderate increase in afterload led to a substantial increase in circulatory work values
Blanco et al. (2013) [54]	AR, cerebral aneurysm	CL	0D TVE	0D (nonideal diode)	1D 128 SA + 3-element WK peripheral circulation + 0D resistance-inductance-capacitance venous and pulmonary circulation (3D CFD cerebral aneurysm)	Generic data (literature-derived)	Patient-specific records from literature	The hemodynamic response to changes in AV pathological condition was sensitive. WSS and OSI maps remained stable, except in acute conditions. WSS index decreased with worsening of pathology, while OSI increased. Mean residence time of particles decreased with increasing severity of insufficiency
Keshavarz-Motamed et al. (2011) [43]	AS (no, mild, moderate, severe AS), COA	OL	0D TVE LV	0D AV (diode, variable resistor, inductor)	0D COA and systemic circulation	Generic data (typical physiological values)	MRI data through COA (patient with coexistent COA and AS)	AS severity increased LV peak pressure, lengthened ejection time. and delayed peak transvalvular flow rate during ejection. COA severity reduced the proportion of total flow rate crossing it. AS and COA severity increased LV SW. LV SW decreased with increasing AV EOA (AV replacement) and decreasing COA area (COA repair)
Liang et al. (2009) [58]	AS, arterial stenoses	CL	0D TVE	0D pressure−flow relationship	1D 55 SA + 0D peripheral and pulmonary circulation	Generic data (literature-derived)	Echo data around left heart [154]	Global hemodynamic effects of stenoses were location-dependent, with AS and aortic stenosis having pronounced hemodynamic changes. AS notably impacted ventricular dynamics and aortic flow, while aortic stenosis had moderate effects with renal and femoral arterial stenoses had minimal impact
Liang et al. (2009) [57]	Aging	CL	0D TVE	–	1D 55 SA + 0D peripheral and pulmonary circulation	Generic data (literature-derived)	Arterial tonometry and SphygmoCor [155]	Isolated arterial stiffening due to aging caused large increases in ES pressure and PV area, moderate decreases in SV and EF, and minor changes in SW and LV power. Coupled VA stiffening during aging preserved SV and EF and increased ES pressure, SW, PV area, and peak LV power compared to isolated arterial stiffening. Arterial stiffening led to increased aortic systolic HTN and PP in old age due to increased aortic characteristic impedance and premature wave reflection. Aortic dilatation could partly counteract these negative effects
Garcia et al. (2007) [44]	AS, systemic HTN, LVH	OL	0D LV	0D pressure-flow relationship AV	0D 3-element WK SA	Generic data (typical physiological values)	Catheterization data (patient underwent AV replacement)	Systemic HTN strongly affected LVH development in AS patients. Mild-to-moderate AS had a lesser impact on LV wall volume than HTN, while severe AS significantly increased wall volume and impacted LVH
Formaggia et al. (2006) [45]	Aging, atherosclerosis	OL	0D TVE LV	AV (closed/opened)	1D 55 SA + 3-element WK	Generic data (literature-derived [95])	Not mentioned	Uncoupled model (vessel) underestimated reflections in the pathological case; the coupled model (heart-vessel) showed greater sensitivity to variations in arterial stiffness. In young adults, waves moved slowly with late arrival of reflections in diastole, while in older individuals wave speed increased and reflections returned in systole. Arterial obstruction had minimal impact on flow and pressure wave contours of the proximal aorta, but the diseased artery markedly altered the contours
Segers et al. (2002) [61]	AR	CL	0D TVE LV	0D AV (resistor) and MV (resistor and diode)	0D 4-element WK SA	Population-averaged data (cardiac catheterization data from [156])	Catheterization data	Aortic leak severity determined Ea through leak resistance. AV repair would increase Ea assuming all other parameters are constant. LV pump efficiency (SW/PV area) was lower than the theoretical predicted value for a given Ea/Ees, except for simulations with intact AV
Sugimachi et al. (2001) [46]	Arteriosclerosis	OL	0D TVE LV	0D AV (diode)	0D SA	Population-averaged data (cardiac catheterization data from [120])	Not mentioned	Increased arterial reflections due to arterial sclerosis had a mild detrimental effect on LV pump function compared to increased peripheral resistance, mainly due to arterial stiffness rather than increased high-frequency reflections
Segers et al. (2000) [63]	Cardiac and arterial hypertrophy and remodeling	CL	0D TVE LV	0D MV and AV (diode)	0D 4-element WK SA	Population-averaged data (sphygmanometer and echo, literature derived [157])	Sphygmo-manometer and echo data	Vascular stiffening raised PP but not systolic BP alone. Arterial remodeling caused HTN only when combined with increased peripheral resistance. In normal LV, concentric remodeling, concentric hypertrophy, and eccentric hypertrophy with HTN, the cardiac contribution to systolic BP increase was 55%, 21%, 65%, and 108% respectively with remaining arterial changes
Segers et al. (2000) [62]	Aging, HTN, LVH	CL	0D TVE LV	0D MV (resistor)	0D 4-element WK SA	Population-averaged data (literature derived)	Catheterization data	Concentric LVH was an adaptation to increased afterload, where LV wall thickness increased to normalize peak systolic wall stress, and increased preload filled to compensate for impaired diastolic filling and normalized ED wall stress

COA: coarctation of aorta; AS: aortic valve stenosis; HTN: hypertension/hypertensive; LVR: left ventricular remodeling; ATAA: ascending thoracic aortic aneurysm; AR: aortic valve regurgitation; MS: mitral valve stenosis; MR: mitral valve regurgitation; LV: left ventricle/ventricular; LVH: left ventricular hypertrophy; HF: heart failure; OL: open loop; CL: closed loop; EM: electromechanical; MRI: magnetic resonance imaging; TVE: time-varying elastance; LA: left atrium; RA: right atrium; RV: right ventricle; FE: finite-element; CT: computed tomography; CFD: computational fluid dynamics; BC: boundary condition; FSI: fluid–structure interaction; MV: mitral valve; AV: aortic valve; SA: systemic arteries; CTA: computed tomography angiography; TL: transmission line; BP: blood pressure; echo: echocardiography; SV: stroke volume; MR: mitral valve regurgitation; PP: pulse pressure; AIx: augmentation index; WSS: wall shear stress; Zva: valvular arterial impedance; SW: stroke work; EF: ejection fraction; HR: heart rate; ED: end-diastole/diastolic; ES: end-systole/diastolic; MPI: myocardial performance index; Ees: ventricular end-systolic elastance; SVR: systemic vascular resistance; OSI: oscillatory shear index; EOA: effective orifice area; Ea: effective arterial elastance; ST: structured-tree

### Supplementary Information

**Additional file 1: Table S1.** Criteria for literature search in databases. **Table S2.** Criteria for article selection. **Table S3.** Checklist for quality assessment of the included studies. **Table S4.** Quality Assessment of Included Studies. **Table S5.** Summary of model development of included studies. **Table S6.** Summary of applications and main findings of included studies.

## Data Availability

Not applicable.

## Abbreviations

AIx: Augmentation index
AR: Aortic valve regurgitation
AS: Aortic valve stenosis
ATAA: Ascending thoracic aortic aneurysm
AV: Aortic valve
BC: Boundary condition
BP: Blood pressure
CFD: Computational fluid dynamics
CL: Closed loop
COA: Aortic coarctation
CT: Computed tomography
CTA: Computed tomography angiography
Ea: Effective arterial elastance
echo: Echocardiography
ED: End-diastole/diastolic
Ees: Ventricular end-systolic elastance
EF: Ejection fraction
EM: Electromechanical
EOA: Effective orifice area
ES: End-systole/systolic
FE: Finite element
FSI: Fluid–structure interaction
HF: Heart failure
HR: Heart rate
HTN: Hypertension/hypertensive
LA: Left atrium
LV: Left ventricle/ventricular
LVH: Left ventricular hypertrophy
LVR: Left ventricular remodeling
MPI: Myocardial performance index
MR: Mitral valve regurgitation
MRI: Magnetic resonance imaging
MS: Mitral valve stenosis
MV: Mitral valve
NS: Navier–Stokes
ODE: Ordinary differential equation
OL: Open loop
OSI: Oscillatory shear index
PDE: Partial differential equation
PP: Pulse pressure
PWV: Pulse wave velocity
RA: Right atrium
RV: Right ventricle
SA: Systemic arteries/arterial
ST: Structured tree
SV: Stroke volume
SVR: Systemic vascular resistance
SW: Stroke work
TL: Transmission line
TVE: Time-varying elastance
WK: Windkessel
WSS: Wall shear stress
VA: Ventricular–arterial
Zva: Valvulo-arterial impedance
