# Supplementary material for: Simulating impaired left ventricular–arterial coupling in aging and disease: a systematic review
Source: Biomed Eng Online. 2024 Feb 22;23:24. doi: 10.1186/s12938-024-01206-2 (PMC10885508; doi:10.1186/s12938-024-01206-2)
Supplement: Supplementary file 1 — Additional file 1: Table S1. Criteria for literature search in databases. Table S2. Criteria for article selection. Table S3. Checklist for quality assessment of the included studies. Table S4. Quality Assessment of Included Studies. Table S5. Summary of model development of included studies. Table S6. Summary of applications and main findings of included studies. [file 12938_2024_1206_MOESM1_ESM.docx]

**Supplementary Material**

**Table S1: Criteria for Literature Search in Databases.**

| **Criterion** | **Inclusion** | **Exclusion** |
| --- | --- | --- |
| **Document type** | Journal article | Review article, conference proceeding, book, book chapter, book series, editorial material, meeting abstract |
| **Language** | English | Non-English |
| **Publication year** | 2000 - 2022 | < 2000 |
| **Area** | Engineering, Mathematics, Cardiovascular System & Cardiology, Computer Science, Physiology | Others |

**Table S2: Criteria for Article Selection.**

| **Criterion** | **Inclusion** | **Exclusion** |
| --- | --- | --- |
| **Study** | Model-based, complication | Clinical, interventions |
| **Technique** | Numerical simulation | Purely predictive models (machine learning) or statistical analysis |
| **Subject** | Human (Adult) | Animal or human (fetal, infants, children) |
| **Interaction** | Left VA coupling | Right VA coupling |
| **Circulation** | Systemic | Purely focus on pulmonary, cerebral and coronary |
| **Condition** | Aging, systemic HTN, and ventricular-valvular-vascular disease | Pulmonary HTN, diabetes, pregnancy |

Abbreviations used: VA, ventricular-arterial; HTN, hypertension.

**Table S3: Checklist for Quality Assessment of the Included Studies.**

| **Categories** | **Item No.** | **Questions** | **Scoring Procedure** | **Type** |
| --- | --- | --- | --- | --- |
| Objective | 1 | Was the research objective clearly stated? | Yes (score = 1); No (score = 0) | Critical |
| Data | 2 | Are the sources of data used for model development (e.g., setting boundary conditions) and parameterization specified? | Yes (score = 1); No (score = 0) | Critical |
| Modelling Approach | 3 | Was the cardiovascular modelling technique adequately described? | Yes (score = 2);  Refer to previous study (score = 1);  No (score = 0) | Critical |
|  | 4 | Were the model assumptions clearly described and justified? | Yes (score = 1); No (score = 0); | Critical |
|  | 5 | Was the modelled health condition clarified (e.g., patient cohort)? | Yes (score = 1); No (score = 0); | Non-critical |
| Parameterization | 6 | Were the parameters used to populate model frameworks specified? | Yes (score = 1);  No (score = 0) | Critical |
| Uncertainty Assessment | 7 | Were sensitivity analyses (e.g., the sensitivity of the model outputs on model parameters) and stability analyses performed and reported? | Yes (score = 1);  No (score = 0); | Non-critical |
| Simulation | 8 | Were the simulation scenarios and methods clearly described? | Yes (score = 1);  No (score = 0) | Critical |
| Validation | 9 | Were the validation methodology and results clearly described? | Yes (score = 1);  No (score = 0) | Critical |
| Results | 10 | Were the research objectives supported by the results? | Yes (score = 1);  No (score = 0) | Critical |
| Key Findings | 11 | Were the key findings clearly discussed and supported by results from other studies? | Yes (score = 1);  No (score = 0) | Critical |
| Limitations | 12 | Were the limitations of the study clearly described? | Yes (score = 1);  No (score = 0) | Non-critical |
| Conclusion | 13 | Were conclusions drawn from the study clearly stated? | Yes (score = 1);  No (score = 0) | Critical |

**Table S4: Quality Assessment of Included Studies.**

| **Authors** | **Year** | **Question Number** | | | | | | | | | | | | | **Quality** |
| --- | --- | --- | --- | --- | --- | --- | --- | --- | --- | --- | --- | --- | --- | --- | --- |
|  |  | **1** | **2** | **3** | **4** | **5** | **6** | **7** | **8** | **9** | **10** | **11** | **12** | **13** |  |
| Caforio et al. [1] | 2022 | 1 | 1 | 2 | 1 | 1 | 1 | 1 | 1 | 1 | 1 | 1 | 1 | 1 | High |
| Laubscher et al. [2] | 2022 | 1 | 1 | 2 | 1 | 1 | 1 | 0 | 1 | 1 | 1 | 1 | 0 | 1 | High |
| Regazzoni et al. [3] | 2022 | 1 | 1 | 2 | 1 | 1 | 1 | 0 | 1 | 0 | 1 | 1 | 0 | 1 | High |
| Wisneski et al. [4] | 2022 | 1 | 1 | 2 | 1 | 1 | 1 | 0 | 1 | 1 | 1 | 1 | 1 | 1 | High |
| Zuo et al. [5] | 2022 | 1 | 1 | 2 | 1 | 1 | 1 | 0 | 1 | 1 | 1 | 0 | 0 | 1 | Medium |
| Sadeghi et al. [6] | 2022 | 1 | 1 | 2 | 1 | 1 | 1 | 1 | 1 | 1 | 1 | 1 | 1 | 0 | High |
| Manganotti et al. [7] | 2021 | 1 | 1 | 2 | 1 | 1 | 1 | 0 | 1 | 1 | 1 | 1 | 0 | 1 | High |
| Pagoulatou et al. [8] | 2021 | 1 | 1 | 1 | 1 | 1 | 1 | 1 | 1 | 1 | 1 | 1 | 1 | 1 | High |
| Pagoulatou et al. [9] | 2021 | 1 | 1 | 2 | 1 | 1 | 1 | 1 | 1 | 1 | 1 | 1 | 1 | 1 | High |
| Cosentino et al. [10] | 2020 | 1 | 1 | 1 | 1 | 1 | 1 | 0 | 1 | 0 | 1 | 1 | 1 | 1 | High |
| Wisneski et al. [11] | 2020 | 1 | 1 | 2 | 1 | 1 | 1 | 0 | 1 | 1 | 1 | 1 | 1 | 1 | High |
| Heusinkveld et al. [12] | 2019 | 1 | 1 | 1 | 1 | 1 | 1 | 0 | 1 | 0 | 1 | 1 | 1 | 1 | High |
| Syomin et al. [13] | 2019 | 1 | 1 | 2 | 1 | 1 | 1 | 0 | 1 | 1 | 1 | 1 | 1 | 0 | High |
| Gul et al. [14] | 2019 | 1 | 1 | 1 | 0 | 1 | 1 | 1 | 1 | 0 | 1 | 0 | 1 | 1 | Medium |
| Shavik et al. [15] | 2018 | 1 | 1 | 2 | 1 | 1 | 1 | 0 | 1 | 1 | 1 | 1 | 1 | 0 | High |
| Liang et al. [16] | 2018 | 1 | 1 | 2 | 1 | 1 | 1 | 1 | 1 | 1 | 1 | 1 | 1 | 1 | High |
| Pagoulatou et al. [17] | 2017 | 1 | 1 | 1 | 1 | 1 | 1 | 0 | 1 | 1 | 1 | 1 | 1 | 1 | High |
| Maksuti et al. [18] | 2016 | 1 | 1 | 1 | 1 | 1 | 1 | 0 | 1 | 1 | 1 | 1 | 1 | 1 | High |
| Chen et al. [19] | 2016 | 1 | 1 | 2 | 1 | 1 | 1 | 0 | 1 | 1 | 1 | 1 | 1 | 1 | High |
| Inuzuka et al. [20] | 2016 | 1 | 1 | 2 | 1 | 1 | 1 | 0 | 1 | 1 | 1 | 1 | 1 | 1 | High |
| Palau-Caballero et al. [21] | 2016 | 1 | 1 | 2 | 1 | 1 | 1 | 0 | 1 | 0 | 1 | 1 | 1 | 1 | High |
| Guala et al. [22] | 2015 | 1 | 1 | 1 | 1 | 1 | 1 | 0 | 1 | 1 | 1 | 1 | 0 | 1 | High |
| Keshavarz-Motamed et al. [23] | 2014 | 1 | 1 | 1 | 1 | 1 | 1 | 0 | 1 | 1 | 1 | 1 | 0 | 1 | High |
| Veress et al. [24] | 2013 | 1 | 1 | 1 | 0 | 1 | 1 | 0 | 1 | 0 | 1 | 1 | 1 | 1 | Medium |
| Blanco et al. [25] | 2013 | 1 | 1 | 2 | 0 | 1 | 1 | 0 | 1 | 1 | 1 | 1 | 0 | 0 | Low |
| Keshavarz-Motamed et al. [26] | 2011 | 1 | 1 | 2 | 1 | 1 | 1 | 1 | 1 | 1 | 1 | 1 | 1 | 1 | High |
| Liang et al. [27] | 2009 | 1 | 1 | 2 | 1 | 1 | 1 | 0 | 1 | 1 | 1 | 1 | 1 | 1 | High |
| Liang et al. [28] | 2009 | 1 | 1 | 2 | 1 | 1 | 1 | 1 | 1 | 1 | 1 | 1 | 1 | 1 | High |
| Garcia et al. [29] | 2007 | 1 | 1 | 1 | 1 | 1 | 1 | 0 | 1 | 1 | 1 | 1 | 1 | 0 | High |
| Formaggia et al. [30] | 2006 | 1 | 1 | 2 | 1 | 1 | 1 | 0 | 1 | 0 | 1 | 1 | 0 | 1 | High |
| Segers et al. [31] | 2002 | 1 | 1 | 2 | 1 | 1 | 1 | 1 | 1 | 1 | 1 | 1 | 1 | 1 | High |
| Sugimachi et al. [32] | 2001 | 1 | 1 | 2 | 1 | 1 | 1 | 0 | 1 | 0 | 1 | 1 | 1 | 1 | High |
| Segers et al. [33] | 2000 | 1 | 1 | 2 | 1 | 1 | 1 | 0 | 1 | 1 | 1 | 1 | 1 | 1 | High |
| Segers et al. [34] | 2000 | 1 | 1 | 2 | 1 | 1 | 1 | 0 | 1 | 1 | 1 | 1 | 1 | 1 | High |

**Table S5: Summary of Model Development of Included Studies.**

| **Authors** | **Study Aim** | **Model Structure** | **Parameterization** | **Validation** |
| --- | --- | --- | --- | --- |
| Caforio et al. (2022) [1] | To investigate the coupled system’s physiological response to variations in the arterial system affecting pulse wave propagation | **Open loop:**  3D EM patient-specific LV (MRI) + 0D valve dynamics [35] + 1D SA (a segment of human upper thoracic aorta; network of 116 arterial segments) terminated with 3WK | **Patient-specific data:**  (1 patient with AS and COA)  LV parameters based on patient-specific set of in vivo MRI measurements at specific instants of the cardiac cycle for the same subject.  Vascular parameters based on imaging-based measurements of the heart and blood flow and invasive BP measurements. | Quality comparison with MRI clinical data under baseline conditions |
| Laubscher et al. (2022) [2] | To investigate the influence of increasing degrees of AS on a cardiovascular system | **Closed loop:**  0D time-varying elastance heart + 0D (M1) simplified (diode) and (M2) advanced [36], (M3) valve pressure loss and motion valves models + 0D SC & PC (sinuses, arteries, arterioles, capillaries, & veins) | **Population-averaged data:**  The physiological parameters used in valve model 3 were taken from 6 literature for an average human male | Comparison with typical human physiological hemodynamic parameters from literature (systolic and diastolic pressure and volume) |
| Regazzoni et al. (2022) [3] | To simulate the response of the electromechanical model by varying preload, afterload and contractility. | **Closed loop:**  3D EM LV; 0D time-varying elastance LA, RA, RV + 0D non-ideal diode valves + 0D SC & PC | **Generic data:**  Literature-derived parameters | Not mentioned |
| Wisneski et al. (2022) [4] | To investigate the effect of AS on LV biomechanics | **Closed loop:**  3D FE patient-specific LV (CT) + 0D MV & AV + 0D circulatory system (SC & PC) | **Patient-specific data:**  Circulatory system and LV parameters tuned according to echocardiographic and catheterization data | Comparison with patient clinical parameters (LV EF, LV and aortic SBP & DBP, mean and peak pressure gradient across AV) |
| Zuo et al. (2022) [5] | To develop a numerical simulation method that can predict the flow field and energy loss of the LV quickly and accurately | **Closed loop:**  1) 0D time-varying voltage LV, RV, LA + 0D AV, MV, TV, PV (diode & resistor) + 0D SC & PC (arterial & venous) (RLC network)  2) 3D CFD realistic LV (boundary condition based on 0D model in (1)) | **Generic data:**  Literature derived parameters  (a set of patient parameters under healthy conditions introduced in Avanzolini et al.’s [37])  Parameterization of LV based on LV flow-time relationship from closed loop system as boundary condition for LV myocardial wall velocity | Comparison with echocardiographic measurements (energy loss in rapid filling & ejection period, atrial systole) and MRI data on MV and AV blood flow velocity (flow velocity in rapid filling & ejection period) |
| Sadeghi et al. (2022) [6] | To investigate the impact of COA and MVD on aortic fluid dynamics | **Closed loop:**  0D time varying elastance LA, LV (double-Hill) + 0D AV, MV (net pressure gradient formulation through valve) + 3D CFD patient-specific thoracic aorta (ascending aorta, aortic branches, descending aorta) (CT) (Lattice Boltzmann method & large eddy simulation) + 0D COA, SC & PC | **Patient-specific data:**  (3 patients with COA and MVD)  Parameters obtained or estimated using Doppler echocardiography and sphygmomanometer measurements. | Validated against clinical Doppler echocardiography velocity magnitudes, cardiac catheterization data, and 4D flow MRI measurements |
| Manganotti et al. (2021) [7] | To develop a complete heart-circulation model compatible with thermodynamics hence stable numerically and informative physiologically. | **Open loop:**  0D reduced heart [38] (assuming thick spherical symmetry, representing LV) (Hill-Maxwell rheological model) + 0D valve dynamics (atrioventricular and AV as diode & resistor) + 1D upper thoracic aorta terminated with 3WK | **Generic data:**  The parameters of the aortic model and the inflow boundary condition used were taken from Xiao et al. 2013 [39]. | Not mentioned |
| Pagoulatou et al. (2021) [8] | To offer in silico insights into the acute and long-term impacts of proximal aortic compliance decrease on central hemodynamics | **Open loop:**  0D time-varying elastance LV + 0D simple valve dynamics + 1D SA (103 arterial segments) terminated with 3WK | **Generic data:**  The cardiac properties were chosen based on previous literature’s physiological ranges.  According to reported literature and broadly recognized remodeling mechanisms | Validated previously against in vivo human data (applanation tonometric derived pressure, PC-MRI & echocardiographic derived flow curves) |
| Pagoulatou et al. (2021) [9] | To investigate how sole changes in cardiac contractility might affect central and peripheral hemodynamics | **Open loop:**  0D time-varying elastance LV + 0D simple valve dynamics + 1D SA (103 arterial segments) terminated with 3WK | **Generic data:**  Based on physiological ranges proposed in the literature (average middle-aged male) | Validated previously against in vivo human data (applanation tonometric derived pressure, PC-MRI & echocardiographic derived flow curves) |
| Cosentino et al. (2020) [10] | To assess the change in the WSS and intramural wall stress of patient-specific ATAA models with different degrees of AS | **Open loop:**  0D heart (CircAdapt model: a deformable myocardial wall with interventricular septum motion, cardiac valve hemodynamic 3WK SC & PC) + 3D CFD patient-specific aorta (CTA) (inlet boundary condition: pulsatile inlet flow waveform by 0D model) terminated with 3WK (supra-aortic vessels & descending aorta) | **Patient-specific data:**  (4 patients with ATAA: 2 bicuspid AV, 2 tricuspid AV with aortic dilation in ascending aorta and aortic root)  Based on clinical and echocardiographic data | Comparison with echocardiographic derived transaortic flow velocity for each patient (error < 5%). |
| Wisneski et al. (2020) [11] | To study the influence of AS on LV myofiber stress | **Closed loop:**  3D FE realistic idealized heart [40] + 0D SC & PC (arteries and veins) | **Generic data:**  Parameters were set based on previous published literature represented an average heart in a middle-aged individual | Ventricular material model has been validated by Genet et al. [41] (tagged MRI derived regional strain) |
| Heusinkveld et al. (2019) [12] | To evaluate the influence of variations in LV contractility and arterial stiffness on AIx | **Closed loop:**  (CircAdapt model)  0D 4-chambered heart (sarcomere modified Hill model) + 1D transmission line arterial and venous tree models with 0D peripheral vascular beds (arteriovenous impedance model) | **Generic data:**  Estimations for reference cross-sectional area and vessel length were based on human data given in Westerhof et al. [42] | Comparison with AIx based on pulse wave analysis of clinical data (applanation tonometry) |
| Syomin et al. (2019) [13] | To evaluate the involvement of haemodynamics in the reduction of the LV performance upon stenosis or regurgitation of the aortic or mitral valve | **Closed loop:**  2D axisymmetric FE idealized LV (mechanics with realistic fibre orientation & advanced description of contraction and regulation of cardiac muscle) +  0D valves (diode, resistor, inductor, capacitor) +  0D SC & PC, atria & RV | **Generic data:**  Based on typical values for healthy humans for SBP & DBP in heart chambers and systemic and lung arteries and veins | Comparison with published data including clinical classification of the severity of the valve defects (Valve stenosis: mean pressure gradients and peak flow velocity; Valve regurgitation: regurgitant volume (RV) & fraction (RF)) |
| Gul et al. (2019) [14] | To study the impact of different HRs in a patient having different levels of aortic abnormalities (stenoses, aneurysms) | **Open loop:**  0D time varying elastance LV + 0D AV, MV (diode) + 0D complete SC (122 arterial segments of major arteries) (viscoelastic Voigt electrical model) | **Generic population data:**  Values of parameters for arterial network based on published literature. Parameter values in heart simulation based on healthy individual. | Not mentioned |
| Shavik et al. (2018) [15] | To replicate how changes in geometrical or material parameters of the aorta affect LV and vice versa | **Closed loop:**  3D FE half prolate ellipsoid LV +  3D FE idealized aorta with uniform wall thickness + 0D SC (arteries, veins), LA, AV, MV | **Generic data:**  Literature derived or estimated parameters | Comparison of LV P-V loop and aorta pressure-diameter curve with normal human systemic circulation measurements under physiological condition |
| Liang et al. (2018) [16] | To inspect the interactions between hemodynamic variables and cardiovascular properties under HTN conditions | **Closed loop:**  0D time-varying elastance heart + 1D large arterial model (55 large arteries) coupled with structured-tree models of distal arteries/arterioles + 0D PC, capillaries, and veins | **Population-averaged data:**  Based on literature derived parameters and in vivo data measured in middle-aged subjects (NTN subjects & HTN patients) | Compared with population-averaged in vivo measurements (NTN & HTN) from different literature (BSP, BDP, BPP, BMP, CI, cfPWV) |

| Pagoulatou et al. (2017) [17] | To evaluate the progression of SBP and PP during normal (non-pathological) aging | **Open loop:**  0D time-varying elastance LV + 0D valves + 1D SA (103 arterial segments) terminated with 3WK | **Population-averaged data:**  Based on literature data on the age-associated changes in arterial stiffness, peripheral resistance and cardiac contractility as an input for the simulations. | Comparison of predicted evolution of pressure and augmentation index with age with the curves obtained in published data from large-scale clinical studies (Framingham Heart Study [43], Anglo-Cardiff Collaborate Trail II [44]) |
| --- | --- | --- | --- | --- |
| Maksuti et al. (2016) [18] | To quantify the mechanisms involved in BP changes during normal aging | **Closed loop:**  0D time-varying elastance LV +  0D valves (diodes) + 0D 4WK SA | **Generic data:**  Arterial parameters (resistance & stiffness) based on literature values.  Cardiac parameters computed through physiological rules (compensated hypertrophy, preservation of EDV) | Comparison of model’s aortic SBP & DBP changes with age with population data for brachial pressure from the Framingham Heart Study |
| Chen et al. (2016) [19] | To study pathological alterations in LV and systemic circulation | **Open loop:**  3D FSI patient specific LV (MRI) (valvular and inflow/outflow tracts (assumed passive), active LV region) [45] + 1D SA (24 large arteries) terminated with structured-tree [46, 47] | **Subject-specific and generic data:**  LV and SA models were initially derived from different healthy human subjects through inverse parameter estimation.  Parameter optimization using inverse method for LV model based on in vivo MRI of a healthy volunteer [48]  Literature derived parameters for SA model [47]  Parameter adjustment to match the measurements of the LV subject according to the ED and SVs for the baseline case. | Comparison with published experimental data (physiological measurements of healthy subjects)  (Baseline model)  Supported by clinical observations (Pathological model) |
| Inuzuka et al. (2016) [20] | To examine comprehensively the effect of changes in cardiac systolic and diastolic function, loading conditions, and HRs on MPI | **Closed loop:**  0D modified time-varying elastance heart + 0D SC & PC (arterial & venous system) (modified 3WK impedances using capacitances, proximal characteristic resistances, peripheral resistances) | **Generic data:**  Literature derived parameters (Baseline values for a 70-kg man adapted from previous report (Burkhoff & Tyberg [49])) | Comparison with changes in echocardiographic derived MPI upon pharmacological manipulation (dobutamine & esmolol infusion) |
| Palau-Caballero et al. (2016) [21] | To investigate how both echocardiographic measures of AR severity and the hemodynamic consequences of AR are influenced by LV and aortic stiffness | **Closed loop:**  (CircAdapt model)  0D atrial and ventricular cavities + 0D atrioventricular and ventriculoarterial valves +  0D aorta, pulmonary and peripheral vascular circulation | **Generic data:**  Literature derived or estimated parameters | Comparison with echocardiographic derived flow velocity across AV from patients with AR |
| Guala et al. (2015) [22] | To elucidate how aortic stiffening and remodelling quantitatively impact the complex interplay between forward and reflected backward waves in the arterial network | **Open loop:**  0D time-varying elastance LV +  0D AV dynamics (detailed force balance on leaflets) +  1D arterial tree model (103 arterial segments) terminated with 0D 3WK models of distal vessels | **Generic data:**  Literature derived or estimated parameters (Diameters, tapering rates and topology of the arterial network (48 branches), distal model parameters) | Comparison with arterial tonometric and pulse wave analysis derived SBP, PP, AIx with aging |
| Keshavarz-Motamed et al. (2014) [23] | To present and validate a new index, N-SW that used to measure global hemodynamic load imposed on LV | **Open loop:**  0D time varying elastance LV (Input: MV mean flow rate) +  0D AV (diode, variable resistor, inductor) (AS: instantaneous net pressure gradient across stenotic valve) + 0D SC (Output: central venous pressure) | **Population-averaged data:**  Measured or calculated based on transthoracic Doppler-echocardiography and CMR data. | Comparison with CMR derived LVOT flow waveforms (healthy subject, patient with mild, moderate, severe AS) |
| Veress et al. (2013) [24] | To generate a framework that permits for the communication of pressure and volume values back and forth between different modeling systems | **Closed loop:**  1) 0D time-varying elastance LA & LV + 0D WK SC models in series (aorta, arteries & capillaries, venous return)  2) 3D FE subject-specific LV (boundary conditions based on 0D model in (1)) | **Generic data:**  Literature derived or estimated parameters.  Initial parameters for the NTN systolic optimizations based on a previous LV model.  Parameter optimization of the circulation to the EDP & EDV values produced by an initial run of the FE model. Optimization of the FE model to reproduce the circulatory pressure and volume values at each of the four time points. | Not mentioned |
| Blanco et al. (2013) [25] | To simulate the effect of aortic insufficiency on the local hemodynamics in a cerebral aneurysm | **Closed loop:**  0D time-varying elastance heart +  0D non-ideal diode valves [36] + 1D SA (128 arterial segments) terminated with 0D 3WK peripheral circulation (arteriole & capillary) + 0D RLC venous (venule, vein, inferior cava) & PC (artery, vein); embedded 3D CFD cerebral aneurysm (patient-specific geometry) | **Generic data:**  Literature derived or estimated parameters (geometric and mechanical parameters of arterial segments, peripheral, venous, & PC) | Comparison with patient-specific records pressure and flow rate published in the literature. |
| Keshavarz-Motamed et al. (2011) [26] | To investigate the impact of coexisting AS and COA on LV workload | **Open loop:**  0D time varying elastance LV (Input: MV mean flow rate) + 0D AV (diode, variable resistor, inductor) (AS: instantaneous net pressure gradient across stenotic valve) + 0D COA (Parallel branches: (1) flow towards upper-body that bypassing the COA (upper body resistance); (2) flow crossing COA and directed towards descending aorta (resistance for proximal descending aorta, time-varying elastance, inductance)) + 0D SC (Output: central venous pressure) | **Generic data:**  Based on typical physiologic values | Comparison with MRI derived flow rate through COA (a patient with coexistent COA and AS) |
| Liang et al. (2009) [27] | To investigate the global hemodynamic effects of aortic valvular and arterial stenoses located in different areas | **Closed loop:**  0D elastance-based heart + 0D valves (pressure-flow relationship) (transvalvular pressure drop across AV) +  1D SA (55 largest arteries) +  0D peripheral circulation (capillary, venule, vein), PC | **Generic data:**  Literature derived or estimated parameters (geometrical & elastic parameters of arteries, heart, cardiac valve, peripheral circulation) | Comparison with echocardiographic derived flow rate in the vicinity of left heart [50] |
| Liang et al. (2009) [28] | To investigate the coupled VA hemodynamic modifications during aging | **Closed loop:**  0D time varying elastance heart +  1D SA model (55 large arteries) +  0D peripheral circulation (arteriole, capillary, venule, vein), PC | **Generic data:**  Literature derived or estimated parameters (physiological data of arterial tree for a healthy young adult, heart, peripheral circulation, PC) | Comparison with arterial tonometric derived radial SBP & DBP and SphygmoCor derived aortic systolic pressure [51] |
| Garcia et al. (2007) [29] | To examine the relative effects of valvular and vascular afterloads on LVH | **Open loop:**  0D LV (with Arts’ model [52] describing myofiber stress) + 0D instantaneous transvalvular pressure-flow relationship AV + 0D 3WK SA | **Generic data:**  Based on typical physiologic values, aortic SBP & DBP chosen according to the classification of BP levels of the European Hypertension Society, and from previous study [53] | Validated previously in patients who underwent an AV replacement (catheterization derived LV and aortic pressures, LV volume) [53] |
| Formaggia et al. (2006) [30] | To simulate the physiological characteristics of individuals of different age and pathological condition. | **Open loop:**  0D time varying elastance LV +  AV (closed/open) + 1D SA (55 largest arteries) terminated with 3WK peripheral circulation | **Generic data:**  Parameters specifying mechanical and physical features of each vessel have been taken from Wang and Parker (2004) [54] and has been normalised with respect to the area of the ascending aorta | Not mentioned |
| Segers et al. (2002) [31] | To assess (1) how Ea, calculated as the ratio of LV ESP and SV, relates to arterial properties and leak severity and (2) the validity of Ea/Emax (with Emax the slope of the ES P-V relation) as a heart-arterial coupling parameter in AR. | **Closed loop:**  0D time varying elastance LV + 0D valves (AV: resistors for forward and backward flow; MV: resistor & diode) + 0D 4WK SA | **Population-averaged data:**  Based on cardiac catheterization data  for 3 subgroups of aortic regurgitation patients derived from Devlin et al. [55]  Cardiac parameters obtained or calculated from literature while arterial parameters and AV leakage assumed fixed values or estimated | Comparison with catheterization derived LV systolic pressure, cardiac output, and regurgitation index |
| Sugimachi et al. (2001) [32] | To assess rises in high- and low-frequency arterial reflections concomitant with age-related arterial sclerosis affect LV pump function | **Open loop:**  0D time-varying elastance LV + 0D AV (diode) + 0D SA impedance (taken from [56]) | **Population-averaged data:**  Based on cardiac catheterization data derived from Murgo et al. [56] | Not mentioned |
| Segers et al. (2000) [33] | To compute the individual and combined contributions of both the arterial system and the heart to SBP in HTN | **Closed loop:**  0D time-varying elastance LV + 0D MV & AV (diode) + 0D 4WK SA | **Population-averaged data:**  Hemodynamic data and model parameters for control subjects and HTNs based on sphygmomanometer and echocardiography, taken directly or derived from Ganau et al [57] | Comparison with sphygmomanometer derived SBP and PP, echocardiographic derived SV |
| Segers et al. (2000) [34] | To investigate VA interaction in HTN induced LVH | **Closed loop:**  0D time-varying elastance LV + 0D MV (linear resistance) + 0D 4WK SA | **Population-averaged data:**  Arterial and cardiac parameters for control subjects and HTN patients with concentric hypertrophy were obtained from literature | Comparison with catheterization derived LV pressure & volume and aortic pressure & flow in 3 patients with dilated cardiomyopathy |

Abbreviations used: 3D, three-dimensional; EM, electromechanical; LV, left ventricle/left ventricular; MRI, magnetic resonance imaging; 0D, zero-dimensional; 1D, one-dimensional; SA, systemic arteries; 3WK, three-element Windkessel; EDV, end-diastolic volume; SV, stroke volume; SC, systemic circulation; PC, pulmonary circulation; LA, left atrium; RV, right ventricle; FE, finite-element; CMR, cardiovascular magnetic resonance; CT, computed tomography; MV, mitral valve; AV, aortic valve; TV, tricuspid valve; PV, pulmonary valve; RLC, resistance-inductance-capacitance; CFD, computational fluid dynamics; 4WK, four-element Windkessel; PP, pulse pressure; SBP, systolic blood pressure; DBP, diastolic blood pressure; LVH, left ventricular hypertrophy; Ea, effective arterial elastance; Emax, end-systolic maximum elastance of the ventricle; AS, aortic valve stenosis; AR, aortic valve regurgitation; COA, coarctation of aorta; AIx, augmentation index; P-V, pressure-volume; WSS, wall shear stress; EF, ejection fraction; ED, end-diastole; ES, end-systole; ESP, end systolic pressure; EDP, end-diastolic pressure; BP, blood pressure; HR, heart rate; HTN, hypertension/hypertensive; NTN, normotension/normotensive; VA, ventricular-arterial; LVOT, left ventricular outflow tract; N-SW, normalized LV stroke work; MPI, myocardial performance index; FSI, fluid-structure interaction; BSP, brachial systolic pressure; BDP, brachial diastolic pressure; BPP, brachial pulse pressure; BMP, brachial mean pressure; CI, cardiac index; cfPWV, carotid-femoral pulse wave velocity; ATAA; ascending thoracic aortic aneurysm; PC-MRI, phase contrast MRI; MVD, mixed valvular diseases

**Table S6: Summary of Applications and Main Findings of Included Studies.**

| **Authors** | **Application** | **Simulation Settings** | **Key Findings** |
| --- | --- | --- | --- |
| Caforio et al. (2022) [1] | Aortic stiffening, COA, aging (bifurcations) | 3D LV coupled to **1D single segment aortic model**:   1. Increase vessel wall stiffness described by Young’s modulus 2. 30% stenosis halfway of arterial segment length   3D LV coupled to **1D network of 116 arterial segments**, with arterial model parameters corresponding to:   1. 25-year-old subject 2. 65-year-old subject | - “Stiffening of the arterial vessel is associated with an increase in ESV at unchanged EDV, leading to a reduction of SV. - “Increase in aortic stiffness with stenosis leads to an increase in peak pressure and a variation in the pressure profile, a reduction in SV caused by an increase of the ESV.” - “Increase in aortic stiffness and systemic vascular resistance (SVR) associated with healthy ageing is reflected in an augmentation of the peak pressure and a variation in the pressure profile, a reduction in SV caused by an increase in ESV.” |
| Laubscher et al. (2022) [2] | AS | Increase degrees of AS severity (a reduction in maximum opening flow area of the valve) by:   1. Model 1: decreasing area opening ratio 2. Model 2 & 3: decreasing maximum opening angles | - The proposed M3 valve model predicts higher pressure drops at more severe degrees of AS when compared to the results of the M1 and M2 models from literature. |
| Regazzoni et al. (2022) [3] | HTN | 1. Preload: changing ventricles preload (EDP) by modifying atrial contractility value 2. Afterload: changing resistance of the arterial circulation 3. Myocardial contractility: changing atrial or ventricular contractility (elastance) | - “The larger the atrial contractility, the more blood is injected in the ventricle, thus increasing preload and SV.” - “Increase of the arterial resistance yields larger values of both the AV opening pressure and the maximal LV pressure (hypertensive effect).” - “An increase in myocardial contractility generates an increase of both maximal LV pressure and SV.” |
| Wisneski et al. (2022) [4] | LFLG AS | Based on single exact patient data | - “Compared to idealized LV geometry and normal ventricular function, reduced LV stress, an initial observation of globally reduced LV stress, was quantified using patient-specific LV model in a case of LFLG AS.” |
| Zuo et al. (2022) [5] | Hypertensive myocardial hypertrophy | 1. Control group (healthy people): normal LV model and normal BP curve (LV flow-time curve) obtained by SIMULINK closed-loop network model simulation. 2. Non-LVH (NLVH) group: closed-loop simulated HTN curve (LV flow-time curve) and normal LV model 3. LVH group: HTN curve (LV flow-time curve) and LV model of myocardial hypertrophy | - “Myocardial hypertrophy associated with HTN had a significant influence on the flow domain. A change in the blood flow made the vortex distribution of the LV in the rapid systole and ejection periods abnormal and not fully developed, leading to higher energy loss, a sharply decreasing blood flow velocity, and a low cardiac EF.” - “The energy loss and velocity distribution of the HTN normal LV group were basically the same as those of the NTN normal LV control group; the values of the two characteristic parameters were slightly greater than those of the control group.” |
| Sadeghi et al. (2022) [6] | COA; MVD | Based on 3 exact patient data:  Varying EOA of MS, MR, AS, AR, and normal valves | - “AR and MR, when coexistent with COA substantially alter the velocity magnitude downstream of the COA, create transitional to turbulent flow downstream of COA and may lead to significant progression of the disease at the COA region.” |
| Manganotti et al. (2021) [7] | Aging | Increase parameter β (surrogate of arterial wall stiffness), adjusting total arterial resistance (TAR) based on increasing pressure, decrease total arterial compliance (TAC):   1. Uncoupled aortic model with imposed inlet flow 2. Fully coupled heart-circulation model | - “Higher systolic peak, lower DBP, and a small increase in wave propagation speed (the dicrotic notch is anticipated)” - “Pressure curve obtained with coupled model is more physiological (a trend towards the merging of the pressure systolic peak and the dicrotic peak).” - “In the ageing case with the uncoupled aortic model, it is possible to observe a double reflection that does not reflect the arterial response to ageing.” |
| Pagoulatou et al. (2021) [8] | LVR, HTN | Aortic banding induced HTN was induced by decreasing TAC of proximal aorta  LV remodelling was simulated by increasing end-systolic elastance and diastolic stiffness parameter, filling pressure was tuned to conserved SV | - “Reduction of proximal aortic compliance leads acutely to HTN, with an increase in the aortic SBP and PP, which is sustained yet slightly alleviated after LV remodelling.” - “Primary mechanism for the increase in PP due to banding is an increase in the forward wave amplitude, which is even more enhanced after LV remodelling.” - “After LV remodelling, stiffer heart causes the forward pressure wave alters its shape, adopting a pronounced upstroke and an earlier peak.” - “LV remodelling is the main cause of the transformation of pressure waveform from old to young phenotype.” |
| Pagoulatou et al. (2021) [9] | Cardiac inotropy | Varying LV contractility by setting end-systolic elastance (Ees) | - “An increase in cardiac contractility alone, with no concomitant change in arterial properties, alters the shape of the forward pressure wave, which, consequently, changes central and peripheral pulse phenotypes.” - “Indices based on the pressure waveform, like AIx, cannot be assumed to reflect only arterial properties. Cardiac contractility is also an important determinant of central AIx.” |
| Cosentino et al. (2020) [10] | ATAA, AS | ATAA based on 4 patient data.  AS severity was simulated by virtually reducing orifice area to obtain desired peak flow velocity (mild, moderate, severe) | - “LV work derived from P-V loops increased with the severity of AS. Post-stenotic hemodynamic and structural variables markedly increased with AS severity, with WSS showing a 10-fold increase for the most severe AS model as compared to the baseline model with a well-functioning AV.” - “Increase in WSS and maximum principal stress of ATAA wall was associated with pronounced values of Zva as an indicator of LV dysfunction.” |
| Wisneski et al. (2020) [11] | AS | 1. Normal AV 2. Moderate & severe AS: simulated by increasing AV resistance parameter | - “Global LV peak systolic myofiber stress increased progressively with increasing degree of AS whereas end-diastolic stress across all conditions varied minimally.” |
| Heusinkveld et al. (2019) [12] | Cardiac inotropy, vascular aging, combined effect of a change in cardiac & vascular tissue properties | 1. Reduced LV sarcomere shortening velocity 2. Increased arterial stiffness by increasing vessel stiffness coefficient (k) 3. Combined (1) & (2) | - “LV contraction velocity influences AIx as much as increased arterial stiffness.” - “Increased AIx does not necessarily relate to an increase in LV SW.” - “Wave reflection magnitude derived from considering pressure, as well as flow, does qualify as a determinant of LV SW.” |
| Syomin et al. (2019) [13] | AS, AR, MS, MR | By varying orifice area:   1. Stenosis: decrease 2. Regurgitation: increase | - AS: Decrease in maximal orifice area of AV caused increase in the mean and maximal difference between the LV and aortic pressures, both LV EDV and ESV, decrease in SV and EF. - MS: At a constant blood volume, LV EDV and SV decreased significantly. - AR: An increase in RV and RF with an increase in the maximal orifice area of valve. - MR: RV and RF increased with orifice area. |
| Gul et al. (2019) [14] | Aortic stenosis and aneurysm | Varying HRs and aortic diameters (different levels of aortic stenoses (30% (weak), 60% (medium), 90% (strong)) and aneurysms (100%, 200%, 400%))  Aorta is divided into two parts (i) thoracic aorta (nodes: 33, 34, 35, 36, 37) and (ii) abdominal aorta (nodes: 40, 41). | - “Aortic stenosis, especially at nodes 33 and 34 has large impact on both pressure and flow in the SC. For flow, stenoses at nodes 35, 36, 37, 40 and 41 have local impact i.e., impact at the node itself or nearby nodes.” - In the presence of aortic stenoses (aneurysms), node 34 (33) has high impact on pressure and flow as compared to node 33 (34). - With increasing HRs, the sensitivity (impact) on pressure and flow in the SC to the level of stenoses and aneurysms increases. |
| Shavik et al. (2018) [15] | Aortic remodelling (wall thickening & stiffening), LV stiffening | Varying aorta wall thickness, mass fractions of the aorta constituents, LV contractility, LV passive stiffness | - “Increasing aorta wall thickness led to a lower LV EF, a higher peak systolic pressure of the LV and a leftward shift in the aorta pressure-diameter relationship with smaller diameter at ED and ES.” - “Increase in collagen mass fraction (decrease in vascular smooth muscle cells and elastic mass fraction) resulted in a higher peak SBP and a reduced LV EF.” - “Decrease in LV contractility led to lower LV EF, lower aortic systolic and pulse pressures, as well as a reduction in the aorta peak stress during the cardiac cycle.” - “With increasing passive stiffness, LV EF decreases and is accompanied by a corresponding decrease in aortic systolic and pulse pressure, as well as peak stress.” |
| Liang et al. (2018) [16] | HTN, arterial stiffening | Varying parameters:   1. **Cardiac:** heart period, peak systolic elastance of LV (LV contractility) 2. **Macrovascular:** Young’s moduli of elastic and peripheral arteries (effective arterial stiffness) 3. **Microvascular:** arteriolar radius and arteriolar media-to-lumen ratio | - “Heart period, central arterial stiffness and arteriolar radius were the major determinant factors for BP and flow pulsatility indices both in large arteries and microcirculation.” - “Structural normalization of distal vessels alone could not fully account for the selected pressure-lowering effects in the aorta unless the stiffness of central arteries was reduced simultaneously, which highlighted the importance of normalizing central arterial stiffness in the management of HTN.” |
| Pagoulatou et al. (2017) [17] | Aging | Simulate aging by:   1. Increase arterial stiffness parameter (PWV) 2. Cardiac parameters: increase end-diastolic elastance (Eed); increase Ees; decrease HR; increase EDP. | - “Forward wave becomes the major determinant of the increase in central and peripheral SBP and PP with advancing age.” - “The stiffening of the proximal aorta and the resulting augmentation of the forward pressure wave is the major contributor of the systolic pressure augmentation with age.” - “AIx was found to increase steeply in young adults and actually decline after 60 years of age.” |
| Maksuti et al. (2016) [18] | Aging | Simulate aging by:   1. Arterial parameters: decrease arterial compliance, increase vascular resistance, increase characteristic impedance (Zc) 2. Cardiac parameters: increase Ees, increase Eed, increase EDP | - “Not only the arterial system, but also the heart, contributes to the changes in BP during aging.” - “The changes in arterial properties initiate SBP increase, which in turn initiates a cardiac remodelling process that further augments systolic pressure and mitigates the decrease in DBP.” |
| Chen et al. (2016) [19] | Arterial stiffening, rarefaction, LVH, inotropy | Applying to coupled model:   1. Increase material constants (k_3_) by 100% in the large arteries 2. Reduce total number of small vessels by decreasing the radius exponent 3. Increase the myocardium stiffness by doubling the values of material parameters 4. Increase LV contractility increasing T_0_ (coefficient of contractile tension)   Applying (1) and (2) to isolated SA model | - “Arterial stiffening and rarefaction cause higher BP along with higher LV active tension, but with reduced SV.” - “LV stiffening leads to severely impaired pump function with low active tension, SV and low BP.” - “Increased contractility can help the heart to maintain a higher SV but causes an elevated pressure in the circulation.” - “Isolated SC model overestimates peak pressure (up to 7%) and flow rate (up to 20%) when compared to coupled model.” |
| Inuzuka et al. (2016) [20] | HF progression with chronic MR | Reduction in systolic function (Ees) followed by development of diastolic dysfunction (exponent of ED P-V relationship, stressed volume) | - “HR increase resulted in a decrease in EF and an increase in MPI. Reduction in end-systolic elastance decreased EF and increased MPI. Volume overload and ventricular stiffening did not affect EF but paradoxically reduced MPI. Increased afterload due to higher SVR resulted in a decrease in EF and increase in MPI, but afterload increase caused by reduced arterial compliance led to a decrease in both EF and MPI. These MPI characteristics caused paradoxical improvement of MPI during disease progression of chronic HF in a simulation of MR.” |
| Palau-Caballero et al. (2016) [21] | AR, LV & aortic stiffness | Different AR severity coexist with normal LV and aortic stiffness, high LV stiffness, high aortic stiffness, high LV & aortic stiffness by:   1. Increasing effective regurgitant orifice area (ROA) of AV to represent no, mild, moderate, and severe AR) 2. Increasing global LV stiffness 3. Increasing stiffness exponent k | - “Increasing ROA resulted in faster decay of diastolic flow velocity and larger regurgitant blood flow across AV caused shorter pressure half-time (PHT) and larger RV and RF, all indicating higher AR severity.” - “Increasing aortic (LV) stiffness resulted in a larger decline (rise) in diastolic aortic (LV) pressure. Hence, increasing LV and/or aortic stiffness led to faster decay of the transvalvular pressure gradient and, therefore, to faster decay of diastolic flow velocity across AV compared with normal stiffness with the same ROA leads to a shorter PHT (high severity scores) and to a lower RF (low severity scores), as less regurgitant blood volume travelled into LV. - “AR severity scores (ROA, RF, PHT) reflected mean left atrial pressure poorly when variations in tissue properties (LV and/or aortic stiffness) were present.” |
| Guala et al. (2015) [22] | Aging, aortic stiffening & remodelling | Adjusting values of the age-dependent coefficients (remodelling) and stiffening trend based on large data set of cfPWV and location-specific trend from published study | - “Ageing-induced aortic stiffening enhances the first pressure pulse generated at the ventricular-aortic interface during ejection, while remodelling damps it.” - “Although stiffening tends to decrease reflection coefficients at network bifurcations, their remodelling-induced large growth prevails, increasing the total amount of reflection. - “Aortic remodelling undermines the protective wave-trapping mechanism on reflected pressure waves while stiffening enhances it.” - “Aortic stiffening and remodelling exhibit a compensatory effect on the pulse pressure amplification: the former reduces it, while the latter augments it.” - “Both stiffening and remodelling contribute to limit the growth of LV work with age.” |
| Keshavarz-Motamed et al. (2014) [23] | AS | Changes in EOA, aortic cross-sectional area from TTE and CMR data from:   1. Heathy subjects 2. Patients with mild, moderate, severe AS | - “N-SW showed very good correlations with a previously validated index of global hemodynamic load, the Zva, using non-invasive measurements.” - “N-SW was almost independent from variations in the flow rate. N-SW is less flow dependent than Zva.” |
| Veress et al. (2013) [24] | HTN | By systematically increasing peripheral resistance until BP values were achieved without an increase in EF (NTN, mild & moderate HTN) | - “CO, SV increased in the mild and moderate HTN cases compared with the normal model.” - “Even mild HTN can cause a marked increase in total LV wall stress.” - “Relatively mild increases in afterload resulted in substantially increased circulatory work values.” |
| Blanco et al. (2013) [25] | AR, cerebral aneurysm | Regurgitant AV is modelled by increasing the minimum angle the AV is able to reach | - “The sensitivity of the global hemodynamics to changes in the pathological condition of the AV is quite large.” - “WSS and OSI maps do not change significantly, except for the most acute condition. While the value of the WSS index tends to be reduced with the development of the pathology into a more severe condition, the value of the OSI tends to increase.” - “Mean residence time of particles decreases with the increase in the insufficiency severity.” |
| Keshavarz-Motamed et al. (2011) [26] | AS (no, mild, moderate, severe AS), COA | 1. Increase AS severity by decreasing EOA 2. Increase coarctation severity by progressive narrowing of COA (increase % by area) | - Increase in AS severity causes progressively increase in LV peak pressure, lengthen ejection time and peak transvalvular flow rate occurs later during ejection phase. - The proportion of the total flow rate that will cross the COA is significantly reduced with the increasing COA severity. - LV SW increases with increasing severity of AS (rapidly from moderate to severe) and COA (smaller impact than moderate AS). - Increasing AV EOA (AV replacement) and decreasing COA by area (COA repair) will reduce LV SW. |
| Liang et al. (2009) [27] | AS, arterial stenoses | Severity of each stenosis was set to be 85%:   1. AV stenosis: EOA of 0.6 cm^2^ (15% of the normal value of 4 cm^2^) 2. 4 stenoses located in thoracic aorta II (No. 13), abdominal aorta IV (No. 31), right renal artery (No. 28), and right femoral artery (No. 38): length of stenosis (2cm) | - “Global hemodynamic influences of the stenoses depend strongly on their locations in the arterial system, particularly, the characteristics of hemodynamic changes induced by the aortic valvular and aortic stenoses are pronounced.” - “AV stenosis exerts the most pronounced impact on ventricular dynamics (reduce SV by about 10.5%) and aortic flow (peak decreases).” - “Aortic stenoses have fairly moderate influences on ventricular dynamics while renal and femoral arterial stenoses have almost no influence on ventricular dynamics (LV P-V loop).” - “Ankle-brachial indices (ABIs) are not significantly different from the normal value in the cases of AV and renal arterial stenoses, but they are lower than 0.92 in the other arterial stenosis cases. Comparing the ABIs smaller than 0.92 reveals that the ABIs have poor correlation with the stenosis locations.” |
| Liang et al. (2009) [28] | Aging | Adjusting elastic parameters of the aorta to match PWV and aortic radius (R) with age (25, 55, 88 years), total peripheral resistance (TPR) increases linearly by 10% between the ages of 25 and 50 years and remains constant thereafter:   1. Isolated arterial stiffening (cardiac parameters kept constant) 2. Coupled VA stiffening (LV Emax increases with age) | - “Aging-associated isolated arterial stiffening induces marked increases in Pes, PVA; moderate reductions in SV, EF; and minor changes in SW, LV power.” - “Coupled VA stiffening (a constant VA coupling index) during aging perfectly preserves SV and EF; and further increases Pes, SW, PVA, and the peak of LV power increases with age compared with those simulated for isolated arterial stiffening.” - “Increased aortic Zc and premature wave reflection induced by arterial stiffening are two coexistent factors responsible for aortic systolic HTN and increased aortic pulse pressure at old age. In contrast, aortic dilatation can partly counteract the negative influences of arterial stiffening.” |
| Garcia et al. (2007) [29] | AS, systemic HTN, LVH | 1. Increase AS severity by decreasing EOA 2. Increase aortic BP level by adjusting SVR (increase) and TAC (decrease) | - “The presence of concomitant systemic HTN has a major influence on the development of LVH in patients with AS.” - “Mild-to-moderate AS has a minor impact on LV wall volume when compared with HTN.” - “For severe AS, wall volume increases exponentially with increasing AS severity and the impact of AS on LVH becomes highly significant.” |
| Formaggia et al. (2006) [30] | Aging, atherosclerosis | 1. Young’s modulus has been reduced by half in the case of an adolescent individual and doubled for an elder 2. Complete obstruction of the right femoral artery by reducing the network to a set of 53 vessels | - “Standard (uncoupled) treatment of proximal boundary conditions underestimates the reflections in the pathological case. The sensitivity to the variation of arterial stiffness of the model using standard proximal conditions (prescribed LV boundary condition) is significantly lower than in the coupled model.” - “Comparing flow and pressure waves, in the young adult the wave speed is low, and reflections arrive late, i.e., they arrive in diastole back in the aorta. In the older individual wave speed has increased and the reflections return in systole.” - “Arterial obstruction causes little perceptible changes in flow and pressure wave contours of the proximal aorta, while flow and pressure wave contours are markedly altered in the diseased artery.” |
| Segers et al. (2002) [31] | AR | Isolated changes in TPR, aortic leak resistance, and TAC based on values from each patient subgroups applied on simulation of Group I (normal contractility, Emax), Group IIa (impaired LV contractility but normal EF), Group IIb (impaired contractility & EF) models | - “Aortic leak severity expressed as leak resistance was the major determinant of Ea.” - “With all other parameters constant, AV repair (no leak) would increase Ea.” - “For a given Ea/Emax, LV pump efficiency (SW/PVA) was lower than the theoretical predicted value, except for the simulations with intact AV.” |
| Sugimachi et al. (2001) [32] | Arteriosclerosis | 1. Type A (highly sclerotic artery in the aged people) 2. Type C (artery with minimal sclerosis in the young) 3. Subtype A (supersclerotic artery) (decreased compliance and increased high-frequency reflections) by a factor of 2, 4, 8 | - “The detrimental effect of increased in arterial reflections associated with arterial sclerosis on SV (LV pump function) is mild compared with the effect of increases in peripheral resistance and mainly attributable to decreased compliance (arterial stiffness) rather than to increased high-frequency reflections.” |
| Segers et al. (2000) [33] | Cardiac & arterial hypertrophy & remodelling | Isolated changes in model parameters of NTN subjects based on hypertensives with normal LV, concentric remodelling (CR), concentric hypertrophy (CH), or eccentric hypertrophy (EH)):   1. **Systemic arterial parameters:** TPR, TAC 2. **Cardiac parameters:** slope of ES P-V relation (Emax), slope of diastolic P-V relation (Emin), venous filling pressure (Pv) | - “Vascular stiffening alone increases PP without increasing SBP. It is only in combination with an increased peripheral resistance that arterial remodelling can lead to HTN.” - “In HTN patients with a normal LV, the heart is responsible for 55% of the increase in SBP with remaining contribution of arterial changes. In CR, CH, and EH, the cardiac contribution (geometric alterations) to the increase in SBP is 21%, 65%, and 108%, respectively.” |
| Segers et al. (2000) [34] | Aging, HTN, LVH | Arterial changes in HTN (25% decrease in TAC and 40% increase in TPR), with conditions:   1. Cardiac parameters kept constant 2. Peak systolic wall stress (σ_s_) is normalized via an increase in LV wall thickness (via Emax & Emin) 3. σ_s_ is normalized through an increase in LV wall thickness, and Pv is changed to normalize diastolic wall stress | - “Concentric LVH can be explained as a cardiac adaptation pattern to an increased afterload, in which peak systolic wall stress is normalized by increasing LV wall thickness, whereas an increased preload filling pressure compensates for the impaired diastolic filling and normalizes ED wall stress.” |

Abbreviations used: COA, coarctation of aorta; 3D, three-dimensional; LV, left ventricle/left ventricular; ESV, end-systolic volume; EDV, end-diastolic volume; SV, stroke volume; SC, systemic circulation; AV, aortic valve; SW/PVA, ratio of stroke work to pressure-volume area; PP, pulse pressure; SBP, systolic blood pressure; DBP, diastolic blood pressure; LVH, left ventricular hypertrophy; Ea, effective arterial elastance; Emax, end-systolic maximum elastance of the ventricle; AS, aortic valve stenosis; AR, aortic valve regurgitation; MR, mitral valve regurgitation; MS, mitral valve stenosis; AIx, augmentation index; P-V, pressure-volume; WSS, wall shear stress; OSI, oscillatory shear index; EF, ejection fraction; ED, end-diastole; ES, end-systole; BP, blood pressure; ROA, effective regurgitant orifice area; HR, heart rate; HTN, hypertension/hypertensive; NTN, normotension/normotensive; EDP, end-diastolic pressure; LFLG, low flow low gradient; MVD, mixed valvular diseases; EOA, effective orifice area; ATAA; ascending thoracic aortic aneurysm; Zva, valvular arterial impedance; PWV, pulse wave velocity; Eed, end-diastolic elastance; Ees, end-systolic elastance; MPI, myocardial performance index; HF, heart failure; cfPWV, carotid-femoral PWV; CO, cardiac output; VA, ventricular-arterial; SVR, systemic vascular resistance; PHT, pressure half-time; RV, regurgitant volume; RF, regurgitant fraction; Emin, slope of diastolic P-V relation; Pv, venous filling pressure; σs, peak systolic wall stress; CR, concentric remodelling; CH, concentric hypertrophy; EH, eccentric hypertrophy; TAR, total arterial resistance; TPR, total peripheral resistance; TAC, total arterial compliance; Zc, characteristic impedance; SW, stroke work; Pes, end-systolic pressure

**Supplement Bibliography**

[1] F. Caforio, C. M. Augustin, J. Alastruey, M. A. F. Gsell, and G. Plank, "A coupling strategy for a first 3D-1D model of the cardiovascular system to study the effects of pulse wave propagation on cardiac function," *Comput. Mech.,* 2022/07/09 2022, doi: 10.1007/s00466-022-02206-6.

[2] R. Laubscher, J. van der Merwe, J. Liebenberg, and P. Herbst, "Dynamic simulation of aortic valve stenosis using a lumped parameter cardiovascular system model with flow regime dependent valve pressure loss characteristics," *Med. Eng. Phys.,* vol. 106, p. 103838, 2022/08/01/ 2022, doi: <https://doi.org/10.1016/j.medengphy.2022.103838>.

[3] F. Regazzoni, M. Salvador, P. C. Africa, M. Fedele, L. Dedè, and A. Quarteroni, "A cardiac electromechanical model coupled with a lumped-parameter model for closed-loop blood circulation," *J. Comput. Phys.,* vol. 457, p. 111083, 2022/05/15/ 2022, doi: <https://doi.org/10.1016/j.jcp.2022.111083>.

[4] A. D. Wisneski *et al.*, "Left Ventricle Biomechanics of Low-Flow, Low-Gradient Aortic Stenosis: A Patient-Specific Computational Model," (in English), *Frontiers in Physiology,* Brief Research Report vol. 13, 2022-April-06 2022, doi: 10.3389/fphys.2022.848011.

[5] X. Zuo *et al.*, "Co-simulation of hypertensive left ventricle based on computational fluid dynamics and a closed-loop network model," (in English), *Comput. Meth. Programs Biomed.,* Article vol. 216, 2022, Art no. 106649, doi: 10.1016/j.cmpb.2022.106649.

[6] R. Sadeghi, N. Gasner, S. Khodaei, J. Garcia, and Z. Keshavarz-Motamed, "Impact of mixed valvular disease on coarctation hemodynamics using patient-specific lumped parameter and Lattice Boltzmann modeling," (in English), *Int. J. Mech. Sci.,* Article vol. 217, p. 26, Mar 2022, Art no. 107038, doi: 10.1016/j.ijmecsci.2021.107038.

[7] J. Manganotti, F. Caforio, F. Kimmig, P. Moireau, and S. Imperiale, "Coupling reduced-order blood flow and cardiac models through energy-consistent strategies: modeling and discretization," *Adv. Model. Simul. Eng. Sci.,* vol. 8, no. 1, p. 21, 2021/09/28 2021, doi: 10.1186/s40323-021-00206-4.

[8] S. Pagoulatou, D. Adamopoulos, G. Rovas, V. Bikia, and N. Stergiopulos, "Acute and Long-Term Effects of Aortic Compliance Decrease on Central Hemodynamics: A Modeling Analysis," (in English), *Frontiers in Physiology,* Original Research vol. 12, 2021-July-26 2021, doi: 10.3389/fphys.2021.701154.

[9] S. Pagoulatou, D. Adamopoulos, G. Rovas, V. Bikia, and N. Stergiopulos, "The effect of left ventricular contractility on arterial hemodynamics: A model-based investigation," *PLOS ONE,* vol. 16, no. 8, p. e0255561, 2021, doi: 10.1371/journal.pone.0255561.

[10] F. Cosentino *et al.*, "On the severity of aortic stenosis in ascending aortic aneurysm: A computational tool to examine ventricular-arterial interaction and aortic wall stress," (in English), *Mech. Res. Commun.,* Article vol. 110, p. 9, Dec 2020, Art no. 103621, doi: 10.1016/j.mechrescom.2020.103621.

[11] A. D. Wisneski *et al.*, "Impact of Aortic Stenosis on Myofiber Stress: Translational Application of Left Ventricle-Aortic Coupling Simulation," (in English), *Frontiers in Physiology,* Article vol. 11, p. 8, Sep 2020, Art no. 574211, doi: 10.3389/fphys.2020.574211.

[12] M. H. G. Heusinkveld *et al.*, "Augmentation index is not a proxy for wave reflection magnitude: mechanistic analysis using a computational model," *J. Appl. Physiol.,* vol. 127, no. 2, pp. 491-500, 2019, doi: 10.1152/japplphysiol.00769.2018.

[13] F. A. Syomin, M. V. Zberia, and A. K. Tsaturyan, "Multiscale simulation of the effects of atrioventricular block and valve diseases on heart performance," (in English), *Int. J. Numer. Meth. Biomed.,* Article vol. 35, no. 7, p. 20, Jul 2019, Art no. e3216, doi: 10.1002/cnm.3216.

[14] R. Gul and S. Shahzadi, "Beat-to-beat sensitivity analysis of human systemic circulation coupled with the left ventricle model of the heart: A simulation-based study," (in English), *Eur. Phys. J. Plus,* Article vol. 134, no. 7, p. 23, Jul 2019, Art no. 314, doi: 10.1140/epjp/i2019-12673-3.

[15] S. M. Shavik, Z. Jiang, S. Baek, and L. C. Lee, "High Spatial Resolution Multi-Organ Finite Element Modeling of Ventricular-Arterial Coupling," (in English), *Frontiers in Physiology,* Original Research vol. 9, 2018-March-02 2018, doi: 10.3389/fphys.2018.00119.

[16] F. Liang, D. Guan, and J. Alastruey, "Determinant Factors for Arterial Hemodynamics in Hypertension: Theoretical Insights from a Computational Model-Based Study," (in English), *J. Biomech. Eng.,* Article vol. 140, no. 3, 2018, Art no. 031006, doi: 10.1115/1.4038430.

[17] S. Pagoulatou and N. Stergiopulos, "Evolution of aortic pressure during normal ageing: A model-based study," *PLOS ONE,* vol. 12, no. 7, p. e0182173, 2017, doi: 10.1371/journal.pone.0182173.

[18] E. Maksuti, N. Westerhof, B. E. Westerhof, M. Broomé, and N. Stergiopulos, "Contribution of the Arterial System and the Heart to Blood Pressure during Normal Aging – A Simulation Study," *PLOS ONE,* vol. 11, no. 6, p. e0157493, 2016, doi: 10.1371/journal.pone.0157493.

[19] W. W. Chen, H. Gao, X. Y. Luo, and N. A. Hill, "Study of cardiovascular function using a coupled left ventricle and systemic circulation model," *Journal of Biomechanics,* vol. 49, no. 12, pp. 2445-2454, 2016/08/16/ 2016, doi: <https://doi.org/10.1016/j.jbiomech.2016.03.009>.

[20] R. Inuzuka, S. Kuwata, C. Kurishima, F. Liang, K. Sughimoto, and H. Senzaki, "Influence of Cardiac Function and Loading Conditions on the Myocardial Performance Index - Theoretical Analysis Based on a Mathematical Model," (in eng), *Circulation Journal* vol. 80, no. 1, pp. 148-56, 2016, doi: 10.1253/circj.CJ-15-0598.

[21] G. Palau-Caballero, J. Walmsley, J. Gorcsan, J. Lumens, and T. Delhaas, "Abnormal Ventricular and Aortic Wall Properties Can Cause Inconsistencies in Grading Aortic Regurgitation Severity: A Computer Simulation Study," (in English), *J. Am. Soc. Echocardiogr.,* Article vol. 29, no. 11, pp. 1122-+, Nov 2016, doi: 10.1016/j.echo.2016.07.015.

[22] A. Guala, C. Camporeale, and L. Ridolfi, "Compensatory Effect between Aortic Stiffening and Remodelling during Ageing," (in English), *Plos One,* Article vol. 10, no. 10, p. 14, Oct 2015, Art no. e0139211, doi: 10.1371/journal.pone.0139211.

[23] Z. Keshavarz-Motamed *et al.*, "Non-Invasive Determination of Left Ventricular Workload in Patients with Aortic Stenosis Using Magnetic Resonance Imaging and Doppler Echocardiography," *PLOS ONE,* vol. 9, no. 1, p. e86793, 2014, doi: 10.1371/journal.pone.0086793.

[24] A. I. Veress, G. M. Raymond, G. T. Gullberg, and J. B. Bassingthwaighte, "Left Ventricular Finite Element Model Bounded by a Systemic Circulation Model," *J. Biomech. Eng.,* vol. 135, no. 5, 2013, doi: 10.1115/1.4023697.

[25] P. J. Blanco and R. A. Feijoo, "A dimensionally-heterogeneous closed-loop model for the cardiovascular system and its applications," (in English), *Med. Eng. Phys.,* Article vol. 35, no. 5, pp. 652-667, May 2013, doi: 10.1016/j.medengphy.2012.07.011.

[26] Z. Keshavarz-Motamed, J. Garcia, P. Pibarot, E. Larose, and L. Kadem, "Modeling the impact of concomitant aortic stenosis and coarctation of the aorta on left ventricular workload," *Journal of Biomechanics,* vol. 44, no. 16, pp. 2817-2825, 2011/11/10/ 2011, doi: <https://doi.org/10.1016/j.jbiomech.2011.08.001>.

[27] F. Liang, S. Takagi, R. Himeno, and H. Liu, "Multi-scale modeling of the human cardiovascular system with applications to aortic valvular and arterial stenoses," (in English), *Medical and Biological Engineering and Computing,* Article vol. 47, no. 7, pp. 743-755, 2009, doi: 10.1007/s11517-009-0449-9.

[28] F. Liang, R. Himeno, and H. Liu, "Biomechanical characterization of ventricular-arterial coupling during aging: A multi-scale model study," *Journal of biomechanics,* vol. 42, pp. 692-704, 05/01 2009, doi: 10.1016/j.jbiomech.2009.01.010.

[29] D. Garcia, P. Pibarot, L. Kadem, and L.-G. Durand, "Respective impacts of aortic stenosis and systemic hypertension on left ventricular hypertrophy," *Journal of Biomechanics,* vol. 40, no. 5, pp. 972-980, 2007/01/01/ 2007, doi: <https://doi.org/10.1016/j.jbiomech.2006.03.020>.

[30] L. Formaggia, D. Lamponi, M. Tuveri, and A. Veneziani, "Numerical modeling of 1D arterial networks coupled with a lumped parameters description of the heart," (in eng), *Comput Methods Biomech Biomed Engin,* vol. 9, no. 5, pp. 273-88, Oct 2006, doi: 10.1080/10255840600857767.

[31] P. Segers, P. Morimont, P. Kolh, N. Stergiopulos, N. Westerhof, and P. Verdonck, "Arterial elastance and heart-arterial coupling in aortic regurgitation are determined by aortic leak severity," (in English), *Am. Heart J.,* Article vol. 144, no. 4, pp. 568-576, Oct 2002, doi: 10.1067/mhj.2002.124398.

[32] M. Sugimachi, T. Shishido, and K. Sunagawa, "Low compliance rather than high reflection of arterial system decreases stroke volume in arteriosclerosis: A simulation," (in English), *Jpn. J. Physiol.,* Article vol. 51, no. 1, pp. 43-51, Feb 2001, doi: 10.2170/jjphysiol.51.43.

[33] P. Segers, N. Stergiopulos, and N. Westerhof, "Quantification of the contribution of cardiac and arterial remodeling to hypertension," (in English), *Hypertension,* Article vol. 36, no. 5, pp. 760-765, Nov 2000, doi: 10.1161/01.Hyp.36.5.760.

[34] P. Segers, N. Stergiopulos, J. J. Schreuder, B. E. Westerhof, and N. Westerhof, "Left ventricular wall stress normalization in chronic pressure-overloaded heart: a mathematical model study," (in English), *American Journal of Physiology-Heart and Circulatory Physiology,* Article vol. 279, no. 3, pp. H1120-H1127, Sep 2000, doi: 10.1152/ajpheart.2000.279.3.H1120.

[35] J. P. Mynard, M. R. Davidson, D. J. Penny, and J. J. Smolich, "A simple, versatile valve model for use in lumped parameter and one-dimensional cardiovascular models," (in English), *Int. J. Numer. Meth. Biomed.,* Article vol. 28, no. 6-7, pp. 626-641, 2012, doi: 10.1002/cnm.1466.

[36] T. Korakianitis and Y. Shi, "Numerical simulation of cardiovascular dynamics with healthy and diseased heart valves," *Journal of Biomechanics,* vol. 39, no. 11, pp. 1964-1982, 2006/01/01/ 2006, doi: <https://doi.org/10.1016/j.jbiomech.2005.06.016>.

[37] G. Avanzolini, P. Barbini, A. Cappello, and A. Cevese, "Time-Varying Mechanical Properties of the Left Ventricle-A Computer Simulation," *IEEE Trans. Biomed. Eng.,* vol. BME-32, no. 10, pp. 756-763, 1985, doi: 10.1109/TBME.1985.325490.

[38] M. Caruel, R. Chabiniok, P. Moireau, Y. Lecarpentier, and D. Chapelle, "Dimensional reductions of a cardiac model for effective validation and calibration," *Biomech. Model. Mechanobiol.,* vol. 13, no. 4, pp. 897-914, 2014/08/01 2014, doi: 10.1007/s10237-013-0544-6.

[39] N. Xiao, J. Alastruey, and C. Alberto Figueroa, "A systematic comparison between 1-D and 3-D hemodynamics in compliant arterial models," (in eng), *Int J Numer Method Biomed Eng,* vol. 30, no. 2, pp. 204-31, Feb 2014, doi: 10.1002/cnm.2598.

[40] B. Baillargeon, N. Rebelo, D. D. Fox, R. L. Taylor, and E. Kuhl, "The Living Heart Project: A robust and integrative simulator for human heart function," *European Journal of Mechanics - A/Solids,* vol. 48, pp. 38-47, 2014/11/01/ 2014, doi: <https://doi.org/10.1016/j.euromechsol.2014.04.001>.

[41] M. K. Rausch, M. Genet, and J. D. Humphrey, "An augmented iterative method for identifying a stress-free reference configuration in image-based biomechanical modeling," *Journal of Biomechanics,* vol. 58, pp. 227-231, 2017/06/14/ 2017, doi: <https://doi.org/10.1016/j.jbiomech.2017.04.021>.

[42] N. Westerhof, F. Bosman, C. J. De Vries, and A. Noordergraaf, "Analog studies of the human systemic arterial tree," *Journal of Biomechanics,* vol. 2, no. 2, pp. 121-143, 1969/05/01/ 1969, doi: <https://doi.org/10.1016/0021-9290(69)90024-4>.

[43] S. S. Franklin *et al.*, "Hemodynamic Patterns of Age-Related Changes in Blood Pressure," *Circulation,* vol. 96, no. 1, pp. 308-315, 1997, doi: doi:10.1161/01.CIR.96.1.308.

[44] C. M. McEniery *et al.*, "Central Pressure: Variability and Impact of Cardiovascular Risk Factors," *Hypertension,* vol. 51, no. 6, pp. 1476-1482, 2008, doi: doi:10.1161/HYPERTENSIONAHA.107.105445.

[45] H. Gao, C. Berry, and X. Luo, "Image-Derived Human Left Ventricular Modelling with Fluid-Structure Interaction," in *Functional Imaging and Modeling of the Heart*, Cham, H. van Assen, P. Bovendeerd, and T. Delhaas, Eds., 2015// 2015: Springer International Publishing, pp. 321-329.

[46] M. S. Olufsen, "Structured tree outflow condition for blood flow in larger systemic arteries," *American Journal of Physiology-Heart and Circulatory Physiology,* vol. 276, no. 1, pp. H257-H268, 1999/01/01 1999, doi: 10.1152/ajpheart.1999.276.1.H257.

[47] M. S. Olufsen, C. S. Peskin, W. Y. Kim, E. M. Pedersen, A. Nadim, and J. Larsen, "Numerical Simulation and Experimental Validation of Blood Flow in Arteries with Structured-Tree Outflow Conditions," *Annals of Biomedical Engineering,* vol. 28, no. 11, pp. 1281-1299, 2000/11/01 2000, doi: 10.1114/1.1326031.

[48] H. Gao, W. G. Li, L. Cai, C. Berry, and X. Y. Luo, "Parameter estimation in a Holzapfel-Ogden law for healthy myocardium," (in eng), *J. Eng. Math.,* vol. 95, no. 1, pp. 231-248, 2015, doi: 10.1007/s10665-014-9740-3.

[49] D. Burkhoff and J. V. Tyberg, "Why does pulmonary venous pressure rise after onset of LV dysfunction: a theoretical analysis," *American Journal of Physiology-Heart and Circulatory Physiology,* vol. 265, no. 5, pp. H1819-H1828, 1993, doi: 10.1152/ajpheart.1993.265.5.H1819.

[50] Y. Sun, M. Beshara, R. J. Lucariello, and S. A. Chiaramida, "A comprehensive model for right-left heart interaction under the influence of pericardium and baroreflex," *American Journal of Physiology-Heart and Circulatory Physiology,* vol. 272, no. 3, pp. H1499-H1515, 1997, doi: 10.1152/ajpheart.1997.272.3.H1499.

[51] C. M. McEniery, Yasmin, I. R. Hall, A. Qasem, I. B. Wilkinson, and J. R. Cockcroft, "Normal Vascular Aging: Differential Effects on Wave Reflection and Aortic Pulse Wave Velocity: The Anglo-Cardiff Collaborative Trial (ACCT)," *J. Am. Coll. Cardiol.,* vol. 46, no. 9, pp. 1753-1760, 2005/11/01/ 2005, doi: <https://doi.org/10.1016/j.jacc.2005.07.037>.

[52] T. Arts, P. H. Bovendeerd, F. W. Prinzen, and R. S. Reneman, "Relation between left ventricular cavity pressure and volume and systolic fiber stress and strain in the wall," *Biophys. J.,* vol. 59, no. 1, pp. 93-102, 1991/01/01/ 1991, doi: <https://doi.org/10.1016/S0006-3495(91)82201-9>.

[53] D. Garcia *et al.*, "A ventricular-vascular coupling model in presence of aortic stenosis," (in English), *American Journal of Physiology-Heart and Circulatory Physiology,* Article vol. 288, no. 4, pp. H1874-H1884, Apr 2005, doi: 10.1152/ajpheart.00754.2004.

[54] J. J. Wang and K. H. Parker, "Wave propagation in a model of the arterial circulation," *Journal of Biomechanics,* vol. 37, no. 4, pp. 457-470, 2004/04/01/ 2004, doi: <https://doi.org/10.1016/j.jbiomech.2003.09.007>.

[55] W. H. Devlin, J. Petrusha, K. Briesmiester, D. Montgomery, and M. R. Starling, "Impact of Vascular Adaptation to Chronic Aortic Regurgitation on Left Ventricular Performance," *Circulation,* vol. 99, no. 8, pp. 1027-1033, 1999, doi: doi:10.1161/01.CIR.99.8.1027.

[56] J. P. Murgo, N. Westerhof, J. P. Giolma, and S. A. Altobelli, "Aortic input impedance in normal man: relationship to pressure wave forms," *Circulation,* vol. 62, no. 1, pp. 105-116, 1980, doi: doi:10.1161/01.CIR.62.1.105.

[57] A. Ganau *et al.*, "Patterns of left ventricular hypertrophy and geometric remodeling in essential hypertension," (in eng), *J Am Coll Cardiol,* vol. 19, no. 7, pp. 1550-8, Jun 1992, doi: 10.1016/0735-1097(92)90617-v.
